# Multivariate Functional Kernel Machine Regression and Sparse Functional Feature Selection

**DOI:** 10.3390/e24020203

**Published:** 2022-01-28

**Authors:** Joseph Naiman, Peter Xuekun Song

**Affiliations:** Department of Biostatistics, University of Michigan, Ann Arbor, MI 48109, USA; jnaiman@umich.edu

**Keywords:** functional principal component analysis, functional predictor, linear mixed-effects model, mobile device, sparse group regularization, wearable device data

## Abstract

Motivated by mobile devices that record data at a high frequency, we propose a new methodological framework for analyzing a semi-parametric regression model that allow us to study a nonlinear relationship between a scalar response and multiple functional predictors in the presence of scalar covariates. Utilizing functional principal component analysis (FPCA) and the least-squares kernel machine method (LSKM), we are able to substantially extend the framework of semi-parametric regression models of scalar responses on scalar predictors by allowing multiple functional predictors to enter the nonlinear model. Regularization is established for feature selection in the setting of reproducing kernel Hilbert spaces. Our method performs simultaneously model fitting and variable selection on functional features. For the implementation, we propose an effective algorithm to solve related optimization problems in that iterations take place between both linear mixed-effects models and a variable selection method (e.g., sparse group lasso). We show algorithmic convergence results and theoretical guarantees for the proposed methodology. We illustrate its performance through simulation experiments and an analysis of accelerometer data.

## 1. Introduction

**Figure 1:**
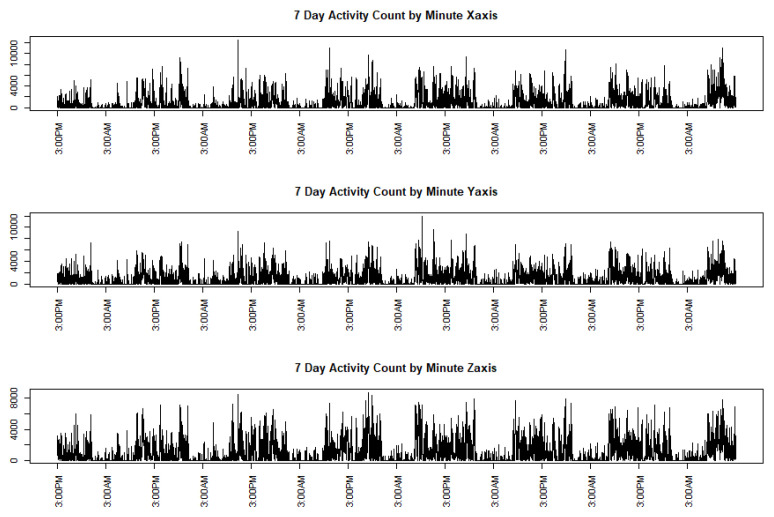
Activity counts over 7 d from a tri-axis (*X*-, *Y*- and *Z*-axis) accelerometer of a subject.

Data captured by mobile devices have lately received much attention in the data science community. Such data are typically recorded at a high frequency, giving rise to an ample volume of information at a very fine scale, and thus present many methodological challenges in statistical modeling and data analyses. In this paper, we plan to utilize the strength of the classical kernel machine method that enjoys fast computing speed via the linear mixed-effects model to deal with such high-frequency data using a functional data analysis approach. The motivation for our proposed framework come from data collected from a tri-axis accelerometer. Accelerometers, worn on the hip or wrist as a way of monitoring physical activity, are becoming more and more common [1,2,3,4]. There are several different accelerometers available such as ActiGraph GT3X+ (ActiGraph, Pensacola, FL, USA) and Actical (Phillips Respironics, Bend, OR). Raw accelerometer data are often collected in high-resolution signals with a sampling frequency ranging from 30–100 Hz. The commercial software on these devices provides activity counts (ACs) [2,4], which are calculated from the raw accelerometer data using proprietary algorithms. As an example from our motivating dataset, Figure 1 displays a three-dimensional time series of ACs per minute, each on one axis, from one subject wearing the GT3X+ over a period of 7 days (d).

Oftentimes, different types of summaries of the tri-axis ACs are suggested in the literature as opposed to the utility of all three raw functionals [5,6,7,8]. These summary-data-based approaches may be regarded as a quick and dirty dimension reduction strategy that comes up with summarized data with computationally manageable volumes, which would be then analyzed by existing methods and software. One concern with the use of summarized data would be the loss of potential fine features that can only be captured in data of high resolution. Recently, some researchers have attempted to use the entire functional AC curve through functional data analysis techniques [6,9,10]. Further details on current methods being used to retrieve and interpret accelerometer data can be found in [11]. Our contribution in this paper pertains to a new framework in that tri-axis accelerometer data are used as three-dimensional correlated functional predictors in an association analysis with a potential health outcome such as the Body Mass Index (BMI). The relationship between physical activities and childhood obesity has long been a central interest of public health sciences, and our new scalar-on-functional regression model can provide some new insights into this important scientific problem.

We begin with a brief review of existing functional data models, the least-squares kernel machine model, and different variable selection techniques, which prelude the framework for this paper.

### 1.1. Functional Regression

There has been much attention in recent years given to functional data analysis (FDA) where either covariates, or response, or both are functional as opposed to scalar in nature [12,13,14,15,16,17]. In this paper, we focused on the methodology that allows us to relate multiple functional covariates to a scalar outcome in a nonlinear way in the presence of other scalar covariates. To proceed, let us introduce some notation. Let $L^{2}\left(T\right)$ be the class of square-integrable functions on a compact set T. This is a separable Hilbert space with inner product <f,$g>:=\int_{T}fg$ for f,$g\in L^{2}\left(T\right)$. Consider a probability space (Ω,F,P), where *Z* denotes a functional random variable that maps into $L^{2}\left(T\right)$, namely $Z:\Omega \mapsto L^{2}\left(T\right)$. Define $L^{2}\left(\Omega \right):=\left\{Z:{\left(\int_{\Omega }{∥Z∥}^{2}dP\right)}^{\frac{1}{2}}<\infty \right\}$, where *P* is a certain probability measure, ${∥Z∥}^{2}=$ <Z, Z>, and assume $Z\in L^{2}\left(\Omega \right)$ in the rest of this paper. For convenience, we also assume that *Z* is mean centered, namely E(Z)=0.

The class of functional linear models (FLM) (e.g., [13,14,15]) is proposed to relate a functional covariate *Z* with a mean-centered scalar outcome *y*, which is also known as scalar-on-functional regression: y=<b,Z>+ϵ, where the error term ϵ is a mean zero random variable uncorrelated with *Z*. An optimal solution of the unknown functional parameter $b\in L^{2}\left(T\right)$ is typically obtained by minimizing the mean-squared error: $\inf_{b\in L^{2}\left(T\right)}E{\left(y-<b,Z>\right)}^{2}$. Moreover, the mean model for the mean-centered scalar *y* takes the form $E\left(y|Z\right)=\int_{T}Z\left(t\right)b\left(t\right)dt$.

As suggested in the literature, we may obtain an optimal estimator of *b* by expanding functional predictor *Z* under certain basis functions. In this paper, we focus on the utility of functional principal component analysis (FPCA) to perform the decomposition of the functional *Z*. By the Karhunen–Loève expansion (e.g., [18,19,20]), we may write $Z\left(t\right)=\sum_{k=1}^{\infty }\sqrt{\varsigma_{k}}\xi_{k}\phi_{k}\left(t\right)$, where $\varsigma_{k}>0$ are the eigenvalues, and the loadings are given by $\xi_{k}:=\frac{1}{\sqrt{\varsigma_{k}}}<Z,\phi_{k}>$. These coefficients satisfy (i) mean zero, $E(\xi_{k})=0$; (ii) variance one, $E(\xi_{k}^{2})=1$; (iii) uncorrelated, $E(\xi_{k}\xi_{j})=0$ for k≠j. Then, the mean model may be rewritten as follows,

(1) $$E\left(y|Z\right)=\sum_{k=1}^{\infty }\beta_{k}\xi_{k},$$

where coefficients $\beta_{k}=<b,\sqrt{\varsigma_{k}}\phi_{k}>$, k=1,···, which are unknown due to the unknown *b*. Equation (1) presents a linear projection of scalar outcome *y* on the space spanned by the standardized principal components (PCs) $\xi_{k}$’s of functional predictor *Z*. On these lines of research, Müller and Yao (2008) proposed a class of functional additive models (FAMs) that extends Equation (1) by allowing a nonparametric form of the projection:

(2) $$E\left(y|Z\right)=\sum_{k=1}^{\infty }f_{k}\left(\xi_{k}\right),$$

where $f_{k}$ is a fully unspecified nonlinear smooth function to be estimated. It is obvious that Müller and Yao’s extension given in (2) takes an additive model on individual coefficient (or feature) components $\xi_{k}$’s. Regularization is often needed for both (1) and (2) in order to deal with these infinite-dimensional unknowns. One of the challenges concerning regularization for (2) lies in the technical treatment in the functional space. Müller and Yao (2008) [21] proposed truncation (or a hard threshold) of the eigenspace to retain only the leading components that explain the majority of the total variation in *Z*. Zhu, Yao, and Zhang (2014) [15] proposed another regularization for the functions $f_{k}$ using the powerful COSSO method [22]. One advantage for this kind of regularization method is that sums of higher-order functional principal components are allowed to be potentially included in the fit model, if they make stronger contributions to the functional relationship than the leading functional principal components. This regularization method [15] begins with an additive model $E\left(y|Z\right)=\sum_{k=1}^{s}f_{k}\left(\xi_{k}\right),$ where *s* represents some initial degrees of truncation to specify the total number of additive components to be considered. Then, COSSO helps simultaneously regularize and select important functional components among the *s* functions $f_{k}$. Although the above discussion is based on a single functional predictor *Z* in mind, it is appealing to extend such a framework with multiple functional predictors for a broad range of problems.

When multiple functional predictors, *say* $Z^{1},\ldots ,Z^{p}$, are considered, it is not clear if the above additive model specification remains suitable to handle the complexity, especially a non-additive relationship (e.g., interactions) may be of interest to understand the association between a scalar outcome and multiple functional predictors. In effect, from both the perspectives of theoretical advances and application needs, relaxing the additive relationship is an important task in functional data analysis. Alternatively, there are some methods (e.g., [16,17]) in the literature that do not use the strategy of decomposing *Z* into its functional components. In this paper, we adopt the framework of kernel machine regression models to extend the methodologies with non-additive relationships between multiple functional predictors and the scalar outcome.

### 1.2. Least-Squares Kernel Machine

Liu, Lin, and Ghosh (2007) [23] proposed a semi-parametric regression model $y_{i}=x_{i}^{⊤}\beta +h\left(z_{i}\right)+\epsilon_{i}$ for subject i=1,…,n, where they used the least-squares kernel machine (LSKM) to analyze multidimensional genetic pathways denoted by a vector $z_{i}$. The key feature of this model is the nonlinear relationship between the outcome $y_{i}$ and a vector of gene expressions $z_{i}$, which is characterized by a nonparametric smooth function *h*. Under the theory of smoothing splines, function *h* is assumed to lie in a reproducing kernel Hilbert space (RKHS), $H_{K}$, generated by a positive-definite kernel function K(·,·). For the ease of exposition, we suppress the bandwidth for the kernel K in the following discussion. Then, both parameter β and function *h* are estimated by maximizing the scaled penalized likelihood function:

(3) $$J\left(h,\beta \right)=-\frac{1}{2}\sum_{i=1}^{n}{\left\{y_{i}-x_{i}^{⊤}\beta -h\left(z_{i}\right)\right\}}^{2}-\frac{1}{2}\lambda_{1}{∥h∥}_{H_{K}}^{2},$$

where $\lambda_{1}>0$ is the tuning parameter and ${∥\cdot ∥}_{H_{K}}$ is the norm of the RKHS. For a function $h\in L^{2}\left(H_{K}\right)$, we have $h\left(\cdot \right)=\sum_{i=1}^{n}\alpha_{i}K\left(\cdot ,z_{i}\right)$. Then, ${∥h∥}_{H_{K}}^{2}=\alpha^{⊤}K\alpha$, where K is an n×n matrix whose (i,j) entry is $K(z_{i},z_{j})$ and $\alpha ={\left(\alpha_{1},\ldots ,\alpha_{n}\right)}^{⊤}$.

It is known in the literature (e.g., [23,24]) that maximizing J(h,β) in (3) turns out to be equivalent to solving the normal equations from the following linear mixed-effects model (LMM): Y=Xβ+h+ϵ, where h is an n×1 vector of random effects with distribution N(0,τK) and an *n*-dimensional vector error term $\epsilon ∼N(0,\sigma^{2}I)$, with $\tau =\lambda_{1}^{-1}\sigma^{2}>0$. One remarkable advantage of solving (3) through the existing numerical procedure of the LMM is most advocated in the literature [25], where we can determine the smoothing parameter $\lambda_{1}$ as part of the estimation of the variance components of the LMM. Therefore, instead of using cross-validation or other information-based tuning methods on $\lambda_{1}$, we can solve simultaneously for all the model parameters in (3), as shown in [23]. Utilizing this numerical strength of the kernel machine regression model, we propose a semi-parametric regression model by incorporating functional principal components of functional predictors (i.e., the $z_{i}$) to evaluate a nonlinear relationship of a scalar outcome with multiple functional covariates in a non-additive way. Assuming that function *h* belongs to an RKHS, we can use existing software packages for solving LMMs to obtain estimates of all model parameters and the smoothing parameter.

### 1.3. Feature Selection

To deal with high-dimensional functional principal components from functional covariates, we invoked the sparse regularization approach in the kernel machine regression model. Note that for both mean models (1) and (2), one needs to truncate the series from the Karhunen–Loève expansion. Regularization helps reduce from an infinite number of terms to a sum of finite terms. To introduce some notations, here we present a brief review on the group lasso (GL) [26], sparse group lasso (SGL) [27], and non-negative garrote [28]. See also the series of work originated by COSSO [22]. Yuan and Lin (2007) [26] proposed the group lasso, which solves the convex optimization problem: $\min_{\beta \in R^{p}}{∥Y-\sum_{\ell =1}^{L}X^{\ell }\beta^{\ell }∥}_{2}^{2}+\lambda \sum_{\ell =1}^{L}{∥\beta^{\ell }∥}_{2}$, where *L* is the total number of groups of covariates and $X^{\ell }$ refers to a subset of covariates associated with group *ℓ*. Friedman, Hastie, and Tibshirani [27] extended the group lasso to allow within-group sparsity, namely SGL, given as $\min_{\beta \in R^{p}}{∥Y-\sum_{\ell =1}^{L}X^{\ell }\beta^{\ell }∥}_{2}^{2}+\lambda \left(1-\delta \right)\sum_{\ell =1}^{L}{∥\beta^{\ell }∥}_{2}+\lambda \delta {∥\beta ∥}_{1}$, where δ∈[0,1]. The additional $\ell_{1}$-norm penalty term on β encourages individual sparsity, while the first penalty targets sparsity at the group level. It is easy to see that group lasso is a special case of the SGL when δ=0.

The non-negative garrote proposed by Breiman (1995) [28] is another useful means of variable selection. It invokes a scaled version of least-squares estimation given by: $arg\min_{d}\frac{1}{2}{∥Y-\tilde{X}d∥}_{2}^{2}+\lambda \sum_{j=1}^{p}d_{j},$ subject to $d_{j}\geq 0,j=1,\ldots ,p$. Here, $\tilde{X}=\left({\tilde{x}}_{1},\ldots ,{\tilde{x}}_{p}\right)$ is an n×p matrix with columns ${\tilde{x}}_{j}=x_{j}{\hat{\beta }}_{j}^{OLS}$, with ${\hat{\beta }}_{j}^{OLS}$ being the least-squares estimates from $arg\min_{\beta }\frac{1}{2}{∥Y-X\beta ∥}_{2}^{2}$ with no constraints. Obviously, estimate ${\hat{d}}_{j}=0$ implies that covariate $x_{j}$ would be excluded from the fit model. Breiman’s formulation that turns a variable selection problem into a parameter estimation problem will be applied for the development of feature selection on functional principal components in this paper.

This paper is organized as follows. Section 2 introduces our proposed high-dimensional kernel machine regression. Section 3 outlines a simple step-by-step algorithm that is used to implement the sparse estimation method. Section 4 concerns asymptotic properties for our proposed sparse kernel machine regression. Section 5 provides simulation results to examine the performance of our method, with comparisons with existing methods. Section 6 illustrates the proposed method by an association analysis of the relationship between the BMI and functional accelerometer data. Section 7 includes our conclusions. The Appendix A contains some key technical details, including the proofs of the theoretical results, while Appendix B presents a discussion on the model identifiability issue.

## 2. Model and Estimation

Consider a regression analysis of a scalar outcome *y* on *p* functional covariates, $Z^{\ell }$, ℓ=1,…,p. Let $z_{i}^{\ell }={\left(\xi_{1}^{\ell },\ldots ,\xi_{s_{\ell }}^{\ell }\right)}_{i}^{⊤}$ be the $s_{\ell }$-element vector of functional principal component (FPC) features from the ith observation of the *ℓ*th functional covariate $Z^{\ell }$, and let ${\vec{z}}_{i}={\left[{\left(z_{i}^{1}\right)}^{⊤},\ldots ,{\left(z_{i}^{p}\right)}^{⊤}\right]}^{⊤}$ be the grand vector of all FPC features from all *p* functional covariates for subject *i*, i=1,…,n. Clearly, the set of FPC features from each functional covariate forms a group, and in total, there are *p* groups with $s=\sum_{\ell =1}^{p}s_{\ell }$ many FPC features and ${\vec{z}}_{i}\in R^{s}$. The high dimensionality of FPC features presents the key methodological challenge in the analysis. We consider the following functional kernel machine regression (FKMR) model:

(4) $$y_{i}=x_{i}^{⊤}\beta +h\left({\vec{z}}_{i}\right)+\epsilon_{i},\;i=1,\cdot \cdot \cdot ,n,$$

where $\beta \in R^{q}$ is a set of parameters for the effects of *q* scalar covariates $x={\left(x_{1},\ldots ,x_{q}\right)}^{⊤}$, $h\in H_{K}$ is an *s*-variate smooth nonparametric function with $H_{K}$ being the functional space generated by a *Mercer kernel*K and error terms $\epsilon_{i}\overset{iid}{∼}N\left(0,\sigma^{2}\right)$. The FKMR model (4) allows for not only nonlinear, but also non-additive relationships with multiple functional covariates $Z^{\ell }$ via their FPC features, ℓ=1,…,p, and a scalar outcome, *y*. The statistical task is to estimate and select important functional covariates that are related to the outcome of interest through regularizing the FPC features within each functional covariate. To proceed, following Beiman’s [28] non-negative garrote method, we here introduce a new *s*-dimensional scaling vector $\gamma \in R^{s}$, $\gamma ={\left(\gamma_{1},\ldots ,\gamma_{s_{1}},\ldots ,\gamma_{s}\right)}^{⊤}$, by which we can set $\gamma \circ {\vec{z}}_{i}={\left(\gamma_{1}\xi_{1}^{1},\ldots ,\gamma_{s_{1}}\xi_{s_{1}}^{1},\ldots ,\gamma_{s}\xi_{s_{p}}^{p}\right)}_{i}^{⊤}$ a new vector of weighted FPC features by γ via the Hadamard product (i.e., elementwise product). Note that γ is grouped and denoted by $\gamma ={\left(\left(\gamma^{1}\right){}^{⊤},\ldots ,{\left(\gamma^{p}\right)}^{⊤}\right)}^{⊤}$ where $\gamma^{\ell }$ is an $s_{\ell }$-element vector of FPC features $z^{\ell }$ of the ℓth functional covariate $Z^{\ell }$. When the element, say $\gamma_{j}$, is equal to zero, the corresponding FPC feature $\xi_{j}$ will not be selected in the set of important FPCs, and moreover, functional covariate $Z^{\ell }$ is excluded from the FKMR model when the entire vector $\left(\gamma^{\ell }\right){}^{⊤}=0$.

We estimate the unknowns in the FKMR model (4), as well as the scaling parameters γ by minimizing the penalized objective function $J_{1}\left(h,\beta ,\gamma \right)$, whose expression is given on the right-hand side of the following Equation (5):

(5) $$\min_{h,\beta ,\gamma }J_{1}\left(h,\beta ,\gamma \right)=\min_{h,\beta ,\gamma }\frac{1}{2n}\sum_{i=1}^{n}{\left\{y_{i}-x_{i}^{⊤}\beta -h\left(\gamma \circ z_{i}\right)\right\}}^{2}+\frac{1}{2}\lambda_{1}{∥h∥}_{H_{K}}^{2}+\lambda_{2}\rho \left(\gamma ;\delta \right),$$

where $\lambda_{1}>0$ and $\lambda_{2}>0$ are two tuning parameters, and penalty ρ(γ;δ) may be specified according to a certain regularization method. For the case of sparse group lasso (SGL), we take $p\left(\gamma ;\delta \right)=\left(1-\delta \right)\sum_{\ell =1}^{p}{∥\gamma^{\ell }∥}_{2}+\delta {∥\gamma ∥}_{1}$, δ∈[0,1]. Typically, δ is predetermined and set to 0.95 or 0.05 depending on the trade-off between group and within-group sparsity, while the factor (1−δ) controls the relative group sparsity to individual sparsity of each functional predictor $Z^{\ell }$. Meanwhile, a large tuning parameter for $\lambda_{2}$ would remove a certain group of FPC features from the FKMR model when all elements in the vector $\gamma^{\ell }$ are zero. Given $h\in H_{K}$, an equivalent optimization to the above (5) can be formulated as follows:

(6) $$\begin{array}{c} \min_{\alpha ,\beta ,\gamma }J_{2}\left(\alpha ,\beta ,\gamma \right)=\min_{\alpha ,\beta ,\gamma }\frac{1}{2n}\sum_{i=1}^{n}{\left\{y_{i}-x_{i}^{⊤}\beta -\sum_{k=1}^{n}\alpha_{k}K\left(\gamma \circ {\vec{z}}_{i},\gamma \circ {\vec{z}}_{k}\right)\right\}}^{2}\\ +\frac{1}{2}\lambda_{1}\alpha^{⊤}K\left(\gamma ;Z\right)\alpha +\lambda_{2}\rho \left(\gamma ;\delta \right), \end{array}$$

where K(γ;Z) is an n×n matrix whose (i,k)th element is ${\left[K\left(\gamma ;Z\right)\right]}_{ik}=K\left(\gamma \circ {\vec{z}}_{i},\gamma \circ {\vec{z}}_{k}\right)$. Lemma 1 below establishes the equivalency of optimization solutions between (5) and (6), which is crucial in our estimation procedure.

**Lemma** **1.** *A solution ($\hat{h}$, $\hat{\beta }$, $\hat{\gamma }$) is a minimizer of (5) if and only if ($\hat{\alpha }$, $\hat{\beta }$, $\hat{\gamma }$) is a minimizer of (6), where $\hat{h}\left(\hat{\gamma }\circ \vec{z}\right)=\sum_{k=1}^{n}{\hat{\alpha }}_{k}K\left(\hat{\gamma }\circ \vec{z},\hat{\gamma }\circ {\vec{z}}_{k}\right)$.*

The proof of Lemma 1 is given in Appendix A.1.

**Theorem** **1** (Existence of optimizers). *If the kernel $K(\cdot ,\gamma \circ \vec{z})$ is continuous with respect to $\gamma \in R^{s}$, then there exists a global minimizer ($\hat{h}$, $\hat{\beta }$, $\hat{\gamma }$) for the optimization problem (5).*

The proof of Theorem 1 is given in Appendix A.3. Note that there may exist multiple optimal minimizers for (5); Theorem 1 ensures only the existence of optimal solutions, but provides no guarantees for uniqueness due to the fact that (5) or (6) is a nonlinear and non-convex optimization problem. It is worth noting that in both (5) and (6), we set the bandwidth for the kernel at a fixed value due to the identifiability issue with respect to the scaling parameters γ. Refer to Appendix B for more detailed discussions on the issue of parameter identifiability.

## 3. Implementation and Algorithm

We propose an iterative algorithm to implement our proposed estimation procedure in which we require the differentiability of the kernel with respect to the scaling factor γ and some additional assumptions presented below in order to ensure algorithmic convergence. One part of the algorithm solving (5) is carried out under fixed γ, where the resulting minimization problem reduces to the equivalent maximization problem in the least-squares kernel machine (3) with the FPC features, ${\vec{z}}_{i}$, being replaced by $\gamma \circ {\vec{z}}_{i}$. As pointed out in Section 1.2, the step of numerical calculation can be easily executed in the same fashion as the solution from the linear mixed model, including the REML estimation of the smoothing parameter $\lambda_{1}$. The other part of the algorithm is performed under fixed α, β and $\lambda_{1}$, where we solve the nonlinear and non-convex optimization problem to update estimates of γ. Lemma 2 below helps us solve for the scaling parameter γ.

**Lemma** **2.** *For fixed (***α***, ***β***, $\lambda_{1}$), minimizing (6) over ***γ*** is equivalent to minimizing over ***γ*** the following objective function:*

(7) $$\frac{1}{2n}{∥F\left(\gamma \right)-\tilde{Y}∥}_{2}^{2}+\lambda_{2}\rho \left(\gamma ;\delta \right),\;\mathrm{for}\;\lambda_{2}>0,$$

*where F(γ)=K(γ;Z)α and $\tilde{Y}=Y-X\beta -\frac{n}{2}\lambda_{1}\alpha$.*

The proof of Lemma 2 is given in Appendix A.2. Linearizing the function F(γ) in (7) leads to an equivalent form:

(8) $$\begin{array}{c} \min_{\gamma }\frac{1}{2n}{∥\tilde{Y}-\sum_{\ell =1}^{p}\nabla_{\gamma }F^{\left(\ell \right)}\left(\tilde{\gamma }\right)\gamma^{\ell }∥}_{2}^{2}+\lambda_{2}\rho \left(\gamma ;\delta \right), \end{array}$$

where $\tilde{Y}=\left(Y-X\beta -\frac{n}{2}\lambda_{1}\alpha \right)-F\left(\tilde{\gamma }\right)+\nabla_{\gamma }F\left(\tilde{\gamma }\right)\tilde{\gamma }$, with $\nabla_{\gamma }F\left(\tilde{\gamma }\right)$ being the gradient of the function F with respect to γ evaluated at $\tilde{\gamma }$ for some $\tilde{\gamma }$, and $\nabla_{\gamma }F^{\left(\ell \right)}\left(\tilde{\gamma }\right)$ being the columns of $\nabla_{\gamma }F\left(\tilde{\gamma }\right)$ associated with the *ℓ*th group of $\gamma^{\ell }$. This is precisely the form of the standard sparse group regularization problem: $\min_{\beta \in R^{p}}\frac{1}{2n}{∥Y-\sum_{\ell =1}^{p}X^{\ell }\beta^{\ell }∥}_{2}^{2}+\lambda_{2}\rho \left(\gamma ;\delta \right).$ This implies that (8) presents a standard sparse group regularization problem with a specific choice of penalty function ρ(γ;δ).

The convergence of the above iterative search algorithm for updating $\tilde{\gamma }$ for fixed (α, β, $\lambda_{1}$) can be justified by the proximal Gauss–Newton method [29]. Readers are referred to [30] for details on the proximal Gauss–Newton method. One of the key assumptions of the proximal Gauss–Newton method is the existence of a local minimizer. This condition is satisfied in the above (8). This is because according to Theorem 1, there exists a global minimizer.

Algorithm 1 summarizes these iterative steps, which is showed to satisfy a descent property: $J_{2}\left(\alpha^{\left(r+1\right)},\beta^{\left(r+1\right)},\gamma^{\left(r+1\right)}\right)$$\leq J_{2}\left(\alpha^{\left(r\right)},\beta^{\left(r\right)},\gamma^{\left(r\right)}\right)$ under the convergence of the proximal Gauss–Newton algorithm for Step 2.2.
**Algorithm 1** An iterative algorithm for optimization in FKMR.1.1Perform FPCA (e.g., the R package fdapace) to extract the functional component features for the *p* functional predictors, and store them in a grand vector for each individual subject ${\vec{z}}_{i}=\left[{\left(z_{i}^{1}\right)}^{⊤},\ldots ,{\left(z_{i}^{p}\right)}^{⊤}\right){]}^{⊤}$, i=1,···,n;1.2Initialize γ to be a vector of ones. which translates to mapping the original component scores to itself. Set up a grid of possible tuning parameters for $\lambda_{1}$ and $\lambda_{2}$, respectively. Set the kernel bandwidth parameter, which may depend on $\lambda_{1}$. For each pair of $\left(\lambda_{1},\lambda_{2}\right)$ from our grid, perform Steps 2.1–2.3 and 3.1 below.2.1At the (r+1)-th step in the algorithm, first solve the LSKM problem with fixed ($\gamma^{\left(r\right)},\lambda_{1}$) (based on a closed-form solution) to update $\beta^{\left(r+1\right)}$ and $\alpha^{\left(r+1\right)}$.2.2Solve the group regularity problem (8) with fixed $\tilde{\gamma }=\gamma^{\left(r\right)}$ and fixed ($\alpha^{\left(r+1\right)}$, $\beta^{\left(r+1\right)}$, $\lambda_{1}$, $\lambda_{2}$) using the r+1 updates from the previous iteration. At this step, the proximal Gauss–Newton algorithm produces an update $\gamma^{\left(r+1\right)}$ at convergence.2.3Repeat Steps 2.1–2.2 until convergence.3.1Perform cross-validation over all pairs of ($\lambda_{1},\lambda_{2}$) to determine the final (α,β,γ).

To speed up Algorithm 1, we propose the following operational schemes that avoid setting up the pairs of ($\lambda_{1}$,$\lambda_{2}$) and performing Step 3.1. Here are a few remarks on the two algorithms. (i) Algorithm 2 depends on good starting values in order to enjoy a fast search. (ii) The main difference between Algorithms 1 and 2 is that $\lambda_{2}$ is fixed in Algorithm 1, while it is changing in Algorithm 2. Some similar algorithms with changing tuning parameters have been proposed in the literature, such as the single index model [31]. (iii) There is no guarantee that both algorithms converge to a global minimizer, and the proximal Gauss–Newton method used in the implementation can only find stationary points. Numerical solvers for the optimization problem in (5) or in (6) indeed remain an open problem in the field of nonlinear and nonconvex optimization.
**Algorithm 2** A fast operational scheme of Algorithm 1.1.Step 2.1 of Algorithm 1 is performed by running the linear mixed model with our initial fixed γ from Step 1.2 of Algorithm 1 to obtained updated values of $\lambda_{1}$, β, and α.2.Step 2.2 is performed with solving the group regularity problem (8) through the Gauss–Newton algorithm using cross-validation-based tuning (e.g., R package oem).3.Rerun Step 2.1 using the updated γ from Step 2.2 to obtain the estimates for β and α.

## 4. Theoretical Guarantees

Our theoretical analysis focuses on the finite-sample $L_{2}$ error bounds for the estimators $\left(\hat{h},\hat{\gamma }\right)$ obtained by (5) or (6). Consequently, we are able to establish the estimation consistency. For simplicity, we set β=0 and consider a general setting of random vectors $z_{1},\ldots ,z_{n}$ so that the FPC features ${\vec{z}}_{1},\ldots ,{\vec{z}}_{n}$ correspond to a special case. Along similar lines as those of [15,32], the estimation consistency is proven in the case of the SGL penalty function. We define a map Γ with an *s*-element vector $\gamma \in R^{s}$, which gives rise to a collection of all scaling map functions: $A=\{\Gamma :R^{s}\mapsto R^{s}|\Gamma \left(z\right)=\gamma \circ z,z\in R^{s}\;\mathrm{and}\;\gamma \in R^{s}\}$. Since Γ is a linear (and bounded) operator, A is a real vector space where $\left(c_{1}\Gamma_{1}+c_{2}\Gamma_{2}\right)\left(z\right)=c_{1}\Gamma_{1}\left(z\right)+c_{2}\Gamma_{2}\left(z\right)$ with any $c_{1},c_{2}\in R$ and $\Gamma_{1},\Gamma_{2}\in A$. To perform a group regularization estimation, we define an SGL penalty by a norm on A for a fixed δ∈[0,1] as follows:

(9) $${∥\Gamma ∥}_{SGL}=\delta \sum_{\ell =1}^{p}{∥\gamma^{\ell }∥}_{2}+\left(1-\delta \right){∥\gamma ∥}_{1}.$$

Consequently, the SGL regularization estimation requires the following constrained optimization:

(10) $$\min_{\Gamma \in A,\;h\in H_{K}}J_{3}\left(\Gamma ,h\right)=\min_{\Gamma \in A,\;h\in H_{K}}{∥Y-h\circ \Gamma ∥}_{n}^{2}+\lambda_{1}{∥h∥}_{H_{K}}^{2}+\lambda_{2}{∥\Gamma ∥}_{SGL},$$

where ${∥Y-h\circ \Gamma ∥}_{n}^{2}=\frac{1}{n}\sum_{i=1}^{n}{\left\{y_{i}-\left(h\circ \Gamma \right)\left(z_{i}\right)\right\}}^{2}$. Lemma 3 below provides the essential finite-sample inequalities that lead to the estimation consistency.

**Lemma** **3** (Basic inequality). *Let $\hat{h}\circ \hat{\Gamma }$ be the minimizer of (10). Let $h_{0}\circ \Gamma_{0}$ be the true function. Then, we have:*

(11) $$J_{3}\left(\hat{\Gamma },\hat{h}\right)\leq 2{\left(\epsilon ,\hat{h}\circ \hat{\Gamma }-h_{0}\circ \Gamma_{0}\right)}_{n}+\lambda_{1}{∥h_{0}∥}_{H_{K}}^{2}+\lambda_{2}{∥\Gamma_{0}∥}_{SGL},$$

*where $2{\left(\epsilon ,\hat{h}\circ \hat{\Gamma }-h_{0}\circ \Gamma_{0}\right)}_{n}=\frac{2}{n}\sum_{i=1}^{n}\epsilon_{i}\left\{\left(\hat{h}\circ \hat{\Gamma }\right)\left(z_{i}\right)-\left(h_{0}\circ \Gamma_{0}\right)\left(z_{i}\right)\right\}$.*

We need the following notation before presenting our theoretical guarantees. Let $N(\delta ,M,P_{n})$ denote the minimal δ covering number of the function set M under the empirical metric $P_{n}$ based on the random vectors $z_{1},\cdots ,z_{n}$. Let $N=N(\delta ,M,P_{n})$ be a shorthand notation. This means that there exist functions $m_{1},\cdots ,m_{N}$ (not necessarily in the set M) such that for every function m∈M, there exists a j∈{1,⋯,N} such that ${∥m-m_{j}∥}_{P_{n}}\leq \delta$, with ${∥m-m_{j}∥}_{P_{n}}:=\sqrt{\frac{1}{n}\sum_{i=1}^{n}{\left\{m\left(z_{i}\right)-m_{j}\left(z_{i}\right)\right\}}^{2}}$. Define the δ-entropy of M for the empirical metric, $P_{n}$, as $H\left(\delta ,M,P_{n}\right):=log\left(N\left(\delta ,M,P_{n}\right)\right)$. Consider a functional space of the form:

$$B=\left\{b:=b\left(h,\Gamma \right)=\frac{h\circ \Gamma -h_{0}\circ \Gamma_{0}}{{∥h∥}_{H_{K}}^{2}+{∥h_{0}∥}_{H_{K}}^{2}+{∥\Gamma ∥}_{SGL}^{2}+{∥\Gamma_{0}∥}_{SGL}^{2}}|h\in H_{K},\Gamma \in A\right\}.$$

We postulate the following assumptions.

**Assumption** **A1.** *The error term $\epsilon ={\left(\epsilon_{1},\ldots ,\epsilon_{n}\right)}^{⊤}$ is uniformly sub-Gaussian; that is, for constants $C_{1}$ and $C_{2}$,*

$$\max_{n\geq 1}\max_{i=1,\cdots ,n}C_{1}^{2}\left[E\left\{exp\left(\frac{{\epsilon_{i}}^{2}}{C_{1}^{2}}\right)\right\}-1\right]\leq C_{2}.$$

*Clearly, the moment condition is bounded below from zero.*

**Assumption** **A2.** *${∥\Gamma_{0}∥}_{SGL}^{2}+{∥h_{0}∥}_{H_{K}}^{2}>0$, and the entropy of space B with respect to the empirical metric $P_{n}$ is bounded as follows:*

$$H\left(\delta ,B,P_{n}\right)\leq C_{3}\delta^{-2\psi },$$

*where $C_{3}$ is some constant and ψ∈(0,1).*

**Assumption** **A3.** *$\sup_{b\in B}{∥b∥}_{P_{n}}\leq C_{4}$ for some constant $C_{4}$.*

**Theorem** **2.** *(Consistency) Under Assumptions 1–3 above, if tuning parameters $\lambda_{1}$ and $\lambda_{2}$ satisfy*

$$\lambda_{2}^{-1}=n^{\frac{1}{1+\psi }}{\left({∥h_{0}∥}_{H_{K}}^{2}+{∥\Gamma_{0}∥}_{SGL}\right)}^{\frac{1-\psi }{1+\psi }},and\lambda_{1}=O_{p}\left(1\right)\lambda_{2},$$

*then we have*

(12) $${∥\hat{h}\circ \hat{\Gamma }-h_{0}\circ \Gamma_{0}∥}_{n}=O_{p}\left(n^{-\frac{1}{2+2\psi }}\right){\left({∥h∥}_{H_{K}}^{2}+{∥\Gamma ∥}_{SGL}\right)}^{\frac{\psi }{1+\psi }},\;and$$

(13) $${∥\hat{h}∥}_{H_{K}}^{2}+{∥\hat{\Gamma }∥}_{SGL}=O_{p}\left(1\right)\left({∥h_{0}∥}_{H_{K}}^{2}+{∥\Gamma_{0}∥}_{SGL}\right).$$

Theorem 2 implies estimation consistency under the right rates for the two tuning parameters $\lambda_{1}$ and $\lambda_{2}$. Due to the potential identifiability issues explained in detail in Appendix B, although the estimator $\left(\hat{h},\hat{\Gamma }\right)$ may not be unique, the sum of $\hat{h}$ and $\hat{\Gamma }$ is not too far away from the sum of the true $h_{0}$ and $\Gamma_{0}$.

**Corollary** **1.** *If the RKHS, $H_{K}$, contains differentiable functions ∇h(z) whose norm ${∥\nabla h(z)∥}_{H_{K}}$ is uniformly bounded for all functions $h\in H_{K}$ and $z\in R^{s}$, then Assumption 2 holds when Theorem 2 is replaced by $H\left(\delta ,H_{K},P_{n}\right)\leq C_{1}\delta^{-2\psi },\;\mathrm{for}\;\mathrm{all}\;\delta \geq 0$.*

The proofs of Theorem 2 and Corollary 1 are given in Appendix A.4 and Appendix A.5, respectively. Often, when we are only interested in a subset of functions in the RKHS (e.g., functions with norm less than one), we can substitute the full space $H_{K}$ in Corollary 1 with the subspace of interest. Refer to [15] or [32], where both considered an RKHS (i.e., Sobolev space) with functions of norm less than or equal to one.

## 5. Simulation Experiments

We performed extensive simulation to investigate the performance of our proposed procedure, including the performance of SGL variable selection and its overall accuracy. Due to the limitations of space, we include results from two simulation experiments in this section, and more results may be found in the first author’s Ph.D. dissertation [30].

### 5.1. Setup

In the evaluation of the performance accuracy, following [15], we used both quasi-$R^{2}$ and adjusted quasi-$R^{2}$ defined as follows:

$$\begin{array}{c} R_{Q}^{2}:=1-\frac{\sum_{i=1}^{n}{\left(y_{i}-\hat{y_{i}}\right)}^{2}}{\sum_{i=1}^{n}{\left(y_{i}-\bar{y_{i}}\right)}^{2}},\;and\;R_{AQ}^{2}:=1-\left(1-R_{Q}^{2}\right)\left(\frac{n-1}{n-(k+1)}\right). \end{array}$$

The latter is known to be appealing for the comparison of the estimation sparsity. There is another performance metric of interest in addition to model accuracy. Performance in variable selection is summarized in terms of the stability measured by sensitivity and specificity for both functional and variable selections under these simulation experiments. Our algorithm uses existing R packages, including emmreml, kspm, and oem.

Specifically, we designed the following two simulation settings.  

Scenario 1: A single functional predictor with sparsity in the FPC features.

Scenario 2: Multiple functional predictors with sparsity in the functional predictors and with sparsity in the FPC features of important functional predictors.  

Each of these two scenarios would be handled using certain suitable penalty functions to address the designed sparsity; for example, in Scenario 2 we used a two-level variable selection penalty (e.g., SGL) to deal with two types of sparsity in the true model. In all analyses, we used the Gaussian kernel $K\left(u,v\right)=\exp\left(-\frac{1}{p}{∥u-v∥}^{2}\right)$ in our estimation, where *p* was set as the number of features, which is equivalent to dividing the γ vector by $\sqrt{p}$. This scaling parameter may be either estimated or set to the number of features to overcome the identifiability issue according to [33], where theoretical justification was given for the use of the number of features for the bandwidth parameter in the case of the Gaussian kernel.

According to [23], due to the difficulty of the graphical display for the estimated *s*-dimensional function h(·) of z, we summarized the goodness-of-fit by regressing the true *h* on the estimated $\hat{h}$, with both being evaluated at the design points. From this concordance regression analysis, we may measure the goodness-of-fit on $\hat{h}$ through the average intercepts, slopes, and *R*-squared (also known as the coefficient of determination) obtained over the number of replications. Clearly, a high-quality fit is reflected by (i) the intercept being close to zero, (ii) the slope being close to one, and (iii) the *R*-squared being close to one. Moreover, we graphically display the estimated function $\hat{h}$ by setting all variables equal to 0.5 except the one of interest over a grid of 100 equally spaced points on the interval [0,1]. Such visualization of the functional estimation at each margin further facilitates the evaluation of the proposed algorithm in addition to the results obtained from the concordance regression analyses.

In all scenarios, we generated 1000 IID functional paths, of which 750 paths were assigned to the training set and 250 paths were assigned to the test set for an external performance evaluation. It is the test set that we used to display the performance accuracy. We used a one-dimensional covariate $x_{i}$ to show the flexibility of our model in a semi-parametric setting, with independent copies of $x_{i}∼N\left(0,1\right)$. We chose the true coefficients in the kernel machine model similar to those given in [23].

### 5.2. Simulation in Scenario 1

In this simple scenario with a single functional predictor, we simulated data from a model with sparsity in its FPC features. To do so, we generated a single functional predictor based on the first 15 eigenbasis of the Fourier basis functions over the interval [0,1]: $Z\left(t\right)=\sum_{j=1}^{15}\sqrt{\varsigma_{j}}\xi_{j}\phi_{j}\left(t\right)$. That is, a functional predictor was created as a linear combination of the 15 basis functions, where $\phi_{j}\left(\cdot \right)$ is the jth Fourier basis function, $\varsigma_{j}$ is the *j*th eigenvalue of *Z*, and $\xi_{j}$ is the *j*th FPC feature that is simulated from a normal distribution detailed as follows.

There were 100 sampled points that were first equally spaced in the interval [0,1] and then varied with certain small deviations drawn from ν∼N(0,0.001). Set $\varsigma_{j}=45\times 0.64^{j}$ and $\xi_{j}∼N\left(0,1\right)$ independently over j=1,…,15. As was done in [17], instead of directly using $\xi_{j}$, we used $\zeta_{j}=\Phi \left(\xi_{j}\right)$, where Φ is the CDF of the standard normal. This resulted in $\vec{z}={\left(\zeta_{1},\ldots ,\zeta_{15}\right)}^{⊤}$. We chose the second, $\zeta_{2}$, and ninth, $\zeta_{9}$, features as important features in the following true nonlinear non-additive model:

$$y_{i}=2x_{i}+20\;\cos\left(2\pi \zeta_{i2}\right)-10\;\sin\left(2\pi \zeta_{i9}\right)+\zeta_{i2}\zeta_{i9}+\epsilon_{i},$$

with $\epsilon_{i}\overset{iid}{∼}N\left(0,1\right)$. FPCA was performed by the R package PACE [34], producing the estimated FPC scores, $\hat{\xi_{j}}$, as well as the estimated eigenvalues, $\hat{\varsigma_{j}}$, which in turn enabled us to compute ${\hat{\zeta }}_{j}$, j=1,…,15.

We applied both LASSO and MCP penalty functions in our implementation, termed as $FKMR_{Lasso}$ and $FKMR_{MCP}$, respectively. We compared the results of our method with the standard linear approach with both LASSO and MCP under the assumption of linear functional relationships, as well as the COSSO method for functional additive regression [15] using the R package COSSO [15,34]. Since the COSSO package is built for nonparametric regression (and not partial linear models), we adopted the backfitting strategy and regressed the residuals with our estimated effect of $x_{i}$ removed.

In addition, we compared our method with an oracle FKMR estimator, called $FKMR^{oracle}$, that assumed the full knowledge of the true $\zeta_{j}$ containing two true nonzero signals, $\zeta_{2}$ and $\zeta_{9}$. We also considered two oracle versions of our proposed algorithm, $FKMR_{Lasso}^{oracle}$ and $FKMR_{MCP}^{oracle}$, both of which used the knowledge of true $\zeta_{j}$ in order to evaluate the performance of the FPCA procedure. This evaluation is important as our proposed procedure can be in principle used in simpler cases that do not involve functional covariates. Note that once we used FPCA to obtain ${\hat{\zeta }}_{j}$ features, our algorithm essentially works in a standard regression setting with the sparsity of covariates. Thus, our proposed procedure can be in principle used in simpler cases with scalar covariates. In Scenario 1, due to the highly nonlinear relationships between the FPC features and the outcome, as expected, the naive linear model performed poorly in terms of both model selection and model consistency. The detailed simulation results for Scenario 1 can be found in the first author’s Ph.D. dissertation [30]. In brief, our proposed method worked well in all aspects. In this setting, COSSO also worked well in terms of model fit, but it tended to select noisy features more frequently than our proposed method, leading to more false positives.

### 5.3. Simulation in Scenario 2

Now, we generated four functional predictors of the form: $Z^{\ell }\left(t\right)=\sum_{j=1}^{9}\sqrt{\varsigma_{j}^{\ell }}\xi_{j}^{\ell }\phi_{j}^{\ell }\left(t\right)$, ℓ=1,…,4, where $\phi_{j}^{\ell }$, $\varsigma_{j}^{\ell }$, and $\xi_{j}^{\ell }$ were set in the same way as those given in Scenario 1. It follows that $\vec{z}={\left(\zeta_{1}^{1},\ldots ,\zeta_{9}^{1},\ldots ,\zeta_{1}^{4},\ldots ,\zeta_{9}^{4}\right)}^{⊤}$, where $\zeta_{j}^{\ell }$ is the *j*th Φ-transformed feature for the *ℓ*th functional covariate. Sparsity was specified as follows: the first and second functional covariates, $Z^{1}$ and $Z^{2}$, were chosen as important signals in which these transformed FPC features, $\left\{\zeta_{1}^{1},\zeta_{3}^{1},\zeta_{4}^{1},\zeta_{2}^{2},\zeta_{7}^{2}\right\}$, are five important features (three features from the $Z^{1}$ and two features from $Z^{2}$) that are related to the outcome:

$$\begin{array}{cc} y_{i}= & \;2x_{i}+\zeta_{i1}^{1}+\zeta_{i3}^{1}+\zeta_{i4}^{1}+\zeta_{i2}^{2}+\zeta_{i7}^{2}+10\cos\left(2\pi \zeta_{i1}^{1}\right)-10{\left(\zeta_{i2}^{2}\right)}^{2}+10{\left(\zeta_{i7}^{2}\right)}^{2}-10{\left(\zeta_{i3}^{1}\right)}^{2}\\ \; & +10\exp\left(-\zeta_{i3}^{1}\right)\zeta_{i4}^{1}-8\sin\left(2\pi \zeta_{i7}^{2}\right)\cos\left(2\pi \zeta_{i3}^{1}\right)+20\zeta_{i1}^{1}\zeta_{i7}^{2}+\epsilon_{i},\;i=1,\ldots ,n, \end{array}$$

where $\epsilon_{i}\overset{iid}{∼}N\left(0,1\right)$. This model specifies both group sparsity (two of the four functional predictors) and within-group sparsity (three of the nine FPC features in $Z^{1}$ and two of the nine FPC features in $Z^{2}$). In addition, we specified non-additive relationships in the true model across multiple functional covariates.

We fit the data using the proposed methods, including $FKMR_{GMCP}^{oracle}$, $FKMR_{Lasso}$, $FKMR_{GLasso}$, $FKMR_{SGL}$, $FKMR_{MCP}$, and $FKMR_{GMCP}$, and the results based on 100 replicates are summarized in Table 1. For comparison, we also fit the simulated data by existing methods, including the linear model (denoted by LM + penalty), COSSO functional additive regression, and the oracle method using the knowledge of true important features in the analysis, as done in the above simulation of Scenario 1. From Table 1 regarding the goodness-of-fit, we see that all of our FKMR estimators outperformed the standard linear estimators in terms of $R_{AQ}^{2}$ among all of our penalty functions, and they outperformed COSSO for penalties that accounted for group sparsity. In the concordance regression analysis, we see that all intercepts were close to zero, all slopes close to one, and all $R^{2}$ close to one, indicating a high goodness-of-fit for functional estimation. COSSO tended to perform on par for penalties that did not account for group sparsity (LASSO and MCP). It is evident that using a group sparsity penalty function (SGL, GLasso, and GMCP) clearly outperformed the methods that did not regularize the grouping of covariates (Lasso and MCP). In addition, our FKMR estimators (except $FKMR_{Lasso}$) performed as well as the oracle estimator $FKMR_{GMCP}^{oracle}$ both in terms of $R_{AQ}^{2}$ and in terms of our estimate of functional *h*. The results also indicated that there were little differences between using a concave (MCP or GMCP) penalty function or using a convex (GLasso or SGL) penalty function.

**Figure 2:**
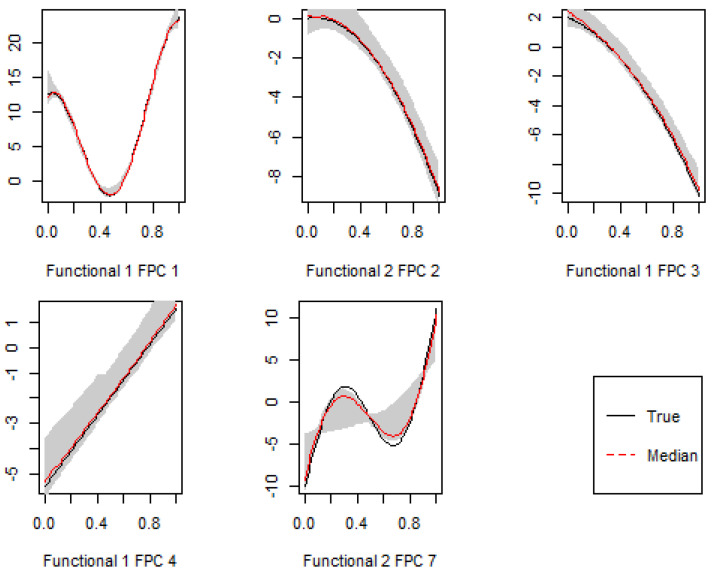
Five marginal estimates of important feature functions with 95% shaded confidence bands evaluated at 100 grid points while holding all other components equal to 0.5 in Scenario 2.

As regards the group sparsity, Table 2 indicates that the all methods had a high sensitivity of detecting functional signals, while the proposed FKMR methods had better specificity than both sparse linear models and COSSO. Concerning the within-group sparsity, it is interesting to note that a bigger difference was seen in terms of what type of penalty function was being used in feature selection. As shown in Table 3 and Table 4, using a general penalty (e.g., Lasso and MCP) that does not take the grouping structure into account tended to under-select important features within a group. COSSO tended to perform well within group sparsity. Moreover, Figure 2 shows that the FKMR method estimated the five signal functions ($Z^{1}$ and $Z^{2}$) well.

## 6. Data Example

**Figure 3:**
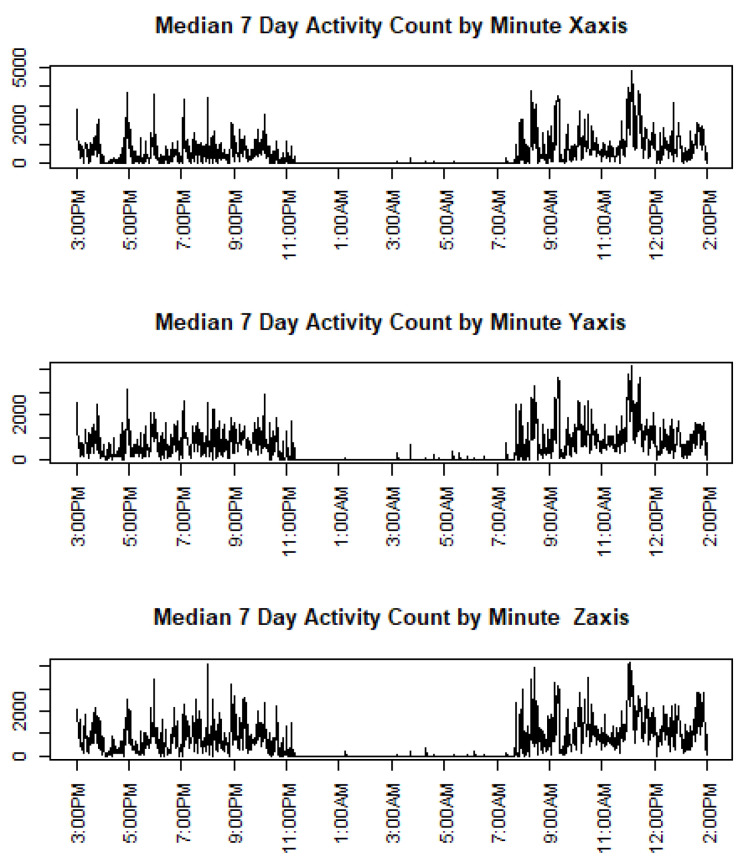
The 24 h minute-by-minute medians of 7 d ACs for one subject.

To show the usefulness of our proposed methodology, we analyzed data of 550 children recruited by the ELEMENTS study [35], who had consent to wear an actigraph (ActiGraph GT3X+; ActiGraph LLC. Pensacola, FL, USA). This wearable was to be placed on their non-dominant wrist for five to seven days with no interruption. The actigraph measured tri-axis accelerometer data sampled at 30 Hz, which captured three different directions of a person’s movement. The BMI was the outcome of interest as it is biomarker of obesity. Sex and age were confounding factors used in the analysis. Due to some missing data, our analysis only included children who wore the device properly for 85% or more over the study period, which resulted in 395 participants, consisting of 189 males and 206 females. Other studies such as [36] have excluded days of accelerometer data with more than five percent missing. The mean ± SD BMI of the study cohort was 21.5 ± 4.1. The mean age of the study participants was 14.3 ± 2.1 y. A more detailed description of the dataset used for this paper can be found in [37]. Our primary interest was to see if the BMI is associated with physical activity in the presence of other covariates, specifically sex and age. We preprocessed the activity counts over the 7 d of wear by taking the median in the 1 min epoch over the entire 7 d of wear. For example, since all the participants started wearing the device at 3 p.m., the first data point for each individual was a median of 7 ACs (each for one day) for the 1 min epoch of 3:00–3:01 p.m. This procedure that takes the medians across the minutes from different days has been considered in other applications such as [36]. See Figure 3 as an example of the resulting time series of medians derived from the AC data displayed in Figure 1.

We applied the following five models, labeled as M0–M4 for convenience, to analyze the data with the 24 h median ACs as functional predictors. Let $\xi_{ij}^{k}$ be the *i*th person’s *k*th FPC score for functional predictor *j*.

M0: Linear model (LM) with only the fixed features: $BMI_{i}∼\beta_{0}+\beta_{1}Age_{i}+\beta_{2}Sex_{i};$
M1: Linear model with SGL penalty (LM+SGL) using the FPCA features: $BMI_{i}∼\beta_{0}+\beta_{1}Age_{i}+\beta_{2}Sex_{i}+\sum_{j=1}^{3}\sum_{k=1}^{s_{k}}\beta_{j}^{k}\xi_{ij}^{k};$
M2: LSKM using the FPCA features: $BMI_{i}∼\beta_{0}+\beta_{1}Age_{i}+\beta_{2}Sex_{i}+h\left(z_{i}\right);$
M3: FKMR model with SGL penalty ($FKMR_{SGL}$) using the FPCA features: $BMI_{i}∼\beta_{0}+\beta_{1}Age_{i}+\beta_{2}Sex+h\left(\gamma \circ z_{i}\right);$
M4: COSSO using the FPCA features: $res\left(BMI_{i}\right)|z_{i}∼\sum_{j=1}^{3}\sum_{k=1}^{s_{k}}f_{ij}\left(\xi_{ij}^{k}\right).$ In order for a direct application of the COSSO R package, we used residuals $res\left(BMI_{i}\right)=BMI_{i}-\hat{\beta_{0}}+\hat{\beta_{1}}Age_{i}+\hat{\beta_{2}}Sex_{i}$ in the COSSO model fit, with ${\hat{\beta }}_{0},{\hat{\beta }}_{1}$ and ${\hat{\beta }}_{2}$ being the estimates of the coefficients from Model M0.

The BMI and age were mean centered and scaled to be a standard deviation of one, so $\beta_{0}$ was absent in the models. Here are some key findings from the data analyses. First, in terms of the goodness-of-fit, Table 5 suggests that M3, i.e., our proposed model FKMR with the SGL penalty, gave the best performance, where the adjusted $R^{2}$ of M3 was nearly twice as big as all the other four models. Second, it is interesting to note that both the COSSO and the $FKMR_{SGL}$ did not select the FPC scores associated with the Z-axis. Third, as shown in Table 6, all of the FPC components chosen by COSSO were also chosen by the $FKMR_{SGL}$. It is worth noting that the linear model together with the SGL penalty selected the highest number of FPC components, yet performed the worst in terms of the model fit.

## 7. Conclusions

In this paper, we proposed a method to model the nonlinear relationship between multiple functional predictors and a scalar outcome in the presence of other scalar confounders. We used the FPCA to decompose the functional predictors for feature extraction and used the LSKM framework to model the functional relationship between the outcome and principal components. We developed a simultaneous procedure to select important functional predictors and important features within selected functionals. We proposed a computationally efficient algorithm to implement our regularization method, which was easily programmed in R with the utility of multiple existing R packages. It should be noted that although we focused on functional regression in this paper, the method proposed can be applied to non-functional predictors. In effect, by using functional principal components, we essentially bypassed the infinite-dimensional problem and worked effectively in a non-functional framework with the FPC features. Through simulation and using data from the ELEMENT dataset, we demonstrated how the FKMR estimator outperformed existing methods in terms of both variable selection and model fit. It should be noted that the existing COSSO method did perform well in terms of variable selection, as shown in Section 5.

A technical issue pertains to identifiability limitations with regard to the bandwidth parameter and to the RKHS estimator. To overcome this, we suggested fixing the bandwidth parameter; see the detailed discussion in Section 3. We established key theoretical guarantees for our proposed estimator. In the case where there are multiple proposed estimators (and thus the identifiability issues arise), the established theoretical properties in Section 4 apply to any of those estimators.

Variable section on functional predictors presents many technical challenges, and there are many methodological problems that remain unsolved. This paper demonstrated a possible framework to regularize estimation with a bi-level sparsity of functional group sparsity and within-group sparsity. In the LSKM paper [23], it was briefly mentioned that if the relationship between the scalar outcome and *p* genetic pathways is additive, we can tweak the model as $y_{i}=x_{i}^{⊤}\beta +h_{1}\left(z_{i}^{1}\right)+\cdots +h_{p}\left(z_{i}^{p}\right)+\epsilon_{i}$ where each $h_{j}$ belongs to its own RKHS. It is easy to extend our method and algorithms to handle this case. For future research, an extension on longitudinal outcomes may be considered via a mixed-effects model $y_{ij}=x_{i}^{⊤}\beta +h\left(z_{ij}\right)+u_{ij}^{⊤}v_{i}+\epsilon_{ij}$ where $u_{ij}^{⊤}v_{i}$ are the random effects. Other useful extensions to the proposed paradigm would be on the lines of generalized linear models and Cox regression models. 

## Figures and Tables

**Table 1:** Goodness-of-fit and the concordance regression for Scenario 2.

Model	$R_{\mathrm{AQ}}^{2}$	β	Reg of *h* on $\hat{h}$		
			Intercept	Slope	$R^{2}$
$FKMR_{Lasso}$	0.830	2.00	−0.062	1.01	0.848
$FKMR_{GLasso}$	0.937	1.99	−0.055	1.01	0.972
$FKMR_{SGL}$	0.928	2.00	−0.051	1.01	0.955
$FKMR_{MCP}$	0.835	2.01	−0.062	1.01	0.856
$FKMR_{GMCP}$	0.935	1.99	−0.056	1.01	0.970
$FKMR_{GMCP}^{oracle}$	0.911	1.99	−0.049	1.01	0.937
COSSO	0.832	–	–	–	–
LM + Lasso	0.453	–	–	–	–
LM + GLasso	0.324	–	–	–	–
LM + SGL	0.450	–	–	–	–
LM + MCP	0.513	–	–	–	–
LM + GMCP	0.307	–	–	–	–

**Table 2:** Sensitivity and specificity of functional selection for Scenario 2.

Model	Selection Frequency			
	$\hat{Z^{1}}$	$\hat{Z^{2}}$	$\hat{Z^{3}}$	$\hat{Z^{4}}$
$FKMR_{Lasso}$	100	100	0	0
$FKMR_{GLasso}$	100	100	4	4
$FKMR_{SGL}$	100	100	0	0
$FKMR_{MCP}$	100	100	0	0
$FKMR_{GMCP}$	100	100	3	4
COSSO	100	100	5	6
LM + Lasso	100	100	19	21
LM + GLasso	94	99	7	8
LM + SGL	100	100	19	18
LM + MCP	100	100	20	19
LM + GMCP	93	99	7	8

**Table 3:** FPC feature selection for signal functional $Z^{1}$ in Scenario 2.

Model	Selection Frequency								
	$\hat{\zeta_{1}^{1}}$	$\hat{\zeta_{2}^{1}}$	$\hat{\zeta_{3}^{1}}$	$\hat{\zeta_{4}^{1}}$	$\hat{\zeta_{5}^{1}}$	$\hat{\zeta_{6}^{1}}$	$\hat{\zeta_{7}^{1}}$	$\hat{\zeta_{8}^{1}}$	$\hat{\zeta_{9}^{1}}$
$FKMR_{Lasso}$	100	1	97	0	0	0	0	0	0
$FKMR_{GLasso}$	100	100	100	100	100	100	100	100	100
$FKMR_{SGL}$	100	21	100	71	26	20	17	16	15
$FKMR_{MCP}$	100	1	99	1	0	0	0	0	0
$FKMR_{GMCP}$	100	100	100	100	100	100	100	100	100
COSSO	100	2	100	93	1	0	0	1	0
LM + Lasso	100	10	100	100	10	8	7	10	5
LM + GLasso	94	94	94	94	94	94	94	94	94
LM + SGL	100	12	100	100	10	8	8	11	5
LM + MCP	100	10	100	100	9	8	9	7	5
LM + GMCP	93	93	93	93	93	93	93	93	93

**Table 4:** FPC feature selection for signal functional $Z^{2}$ in Scenario 2.

Model	Selection Frequency								
	$\hat{\zeta_{1}^{2}}$	$\hat{\zeta_{2}^{2}}$	$\hat{\zeta_{3}^{2}}$	$\hat{\zeta_{4}^{2}}$	$\hat{\zeta_{5}^{2}}$	$\hat{\zeta_{6}^{2}}$	$\hat{\zeta_{7}^{2}}$	$\hat{\zeta_{8}^{2}}$	$\hat{\zeta_{9}^{2}}$
$FKMR_{Lasso}$	0	3	0	0	0	0	100	0	0
$FKMR_{GLasso}$	100	100	100	100	100	100	100	100	100
$FKMR_{SGL}$	16	100	14	7	16	23	100	15	7
$FKMR_{MCP}$	0	11	0	0	0	1	100	0	0
$FKMR_{GMCP}$	100	100	100	100	100	100	100	100	100
COSSO	8	97	5	5	5	15	100	3	3
LM + Lasso	17	100	14	7	16	23	100	15	6
LM + GLasso	99	99	99	99	99	99	99	99	99
LM + SGL	17	100	14	7	16	23	100	15	7
LM + MCP	17	100	13	6	16	23	100	15	8
LM + GMCP	99	99	99	99	99	99	99	99	99

**Table 5:** Goodness-of-fit for the five models used in the data analysis.

Model	Adjusted $R^{2}$
M0: LM	0.07
M1: LM + SGL	0.13
M2: LSKM	0.18
M3: $FKMR_{SGL}$	0.30
M4: COSSO	0.14

**Table 6:** Axis-specific FPC feature selection.

Model	*X*-Axis						*Y*-Axis					*Z*-Axis			
	$\hat{\zeta_{1}^{1}}$	$\hat{\zeta_{2}^{1}}$	$\hat{\zeta_{3}^{1}}$	$\hat{\zeta_{4}^{1}}$	$\hat{\zeta_{5}^{1}}$	$\hat{\zeta_{6}^{1}}$	$\hat{\zeta_{1}^{2}}$	$\hat{\zeta_{2}^{2}}$	$\hat{\zeta_{3}^{2}}$	$\hat{\zeta_{4}^{2}}$	$\hat{\zeta_{5}^{2}}$	$\hat{\zeta_{1}^{3}}$	$\hat{\zeta_{2}^{3}}$	$\hat{\zeta_{3}^{3}}$	$\hat{\zeta_{4}^{3}}$
$FKMR_{SGL}$		✓	✓	✓		✓	✓		✓		✓				
COSSO				✓			✓		✓						
LM + SGL	✓			✓	✓	✓	✓	✓	✓						✓

## Data Availability

The used data of physical activity counts, BMI and demographic variables (sex and age) are available upon request through a formal data request procedure outlined by the ELEMENT Cohort Study. Contact the corresponding author of this paper for the detail.

## Appendix A. Technical Assumptions and Proofs

### Appendix A.1. Proof of Lemma 1

It suffices to show that for any $J_{1}\left(h,\beta ,\gamma \right)$ in (5) we can always find $\alpha \in R^{n}$ such that $J_{1}\left(\tilde{h}=\sum_{i=1}^{n}\alpha_{i}K\left(\cdot ,\gamma \circ {\vec{z}}_{i}\right),\gamma ,\beta \right)\leq J_{1}\left(h,\beta ,\gamma \right)$ where $\tilde{h}$ is the projection of *h* onto the linearly spanned space given by $span\{K\left(\cdot ,\gamma \circ {\vec{z}}_{i}\right),\cdots ,K\left(\cdot ,\gamma \circ {\vec{z}}_{n}\right)\}$. For any *h* we can write $h=h^{⊥}+\tilde{h}\;$ where $h^{⊥}\in span{\left\{K\left(\cdot ,\gamma \circ {\vec{z}}_{1}\right),\cdot \cdot \cdot ,K\left(\cdot ,\gamma \circ {\vec{z}}_{n}\right)\right\}}^{⊥}$. Since $H_{k}$ is a reproducing kernel Hilbert space we can rewrite (5) as follows:

$$\begin{array}{cc} J_{1}\left(h,\gamma ,\beta \right) & =\frac{1}{2n}\sum_{i=1}^{n}{\left\{y_{i}-x_{i}^{⊤}\beta -<h,K\left(\cdot ,\gamma \circ {\vec{z}}_{i}\right)>\right\}}^{2}+\frac{1}{2}\lambda_{1}{∥h∥}_{H_{k}}^{2}+\lambda_{2}\rho \left(\gamma ;\delta \right). \end{array}$$

Since $<h^{⊥},K\left(\cdot ,\gamma \circ {\vec{z}}_{i}\right)>=0$ for every *i*, we obtain

$$\begin{array}{cc} J_{1}\left(h,\gamma ,\beta \right) & =\frac{1}{2n}\sum_{i=1}^{n}{\left\{y_{i}-x_{i}^{⊤}\beta -\sum_{k=1}^{n}\alpha_{k}K\left(\gamma \circ {\vec{z}}_{i},\gamma \circ {\vec{z}}_{k}\right))\right\}}^{2}+\frac{1}{2}\lambda_{1}{∥h^{⊥}+\tilde{h}∥}_{H_{k}}^{2}+\lambda_{2}\rho \left(\gamma ;\delta \right)\\ \; & \geq \frac{1}{2n}\sum_{i=1}^{n}{\left\{y_{i}-x_{i}^{⊤}\beta -\sum_{k=1}^{n}\alpha_{k}K\left(\gamma \circ {\vec{z}}_{i},\gamma \circ {\vec{z}}_{k}\right))\right\}}^{2}+\frac{1}{2}\lambda_{1}{∥\tilde{h}∥}_{H_{k}}^{2}+\lambda_{2}\rho \left(\gamma ;\delta \right)\\ \; & =J_{1}\left(\tilde{h},\gamma ,\beta \right). \end{array}$$

### Appendix A.2. Proof of Lemma 2

The equivalence of forms become clear once we rewrite (6) in the matrix notation. Equation (6) can be written as follows:

(A1) $$\begin{array}{c} \min_{\alpha ,\beta ,\gamma }J_{2}\left(\alpha ,\beta ,\gamma \right)=\min_{\alpha ,\beta ,\gamma }\frac{1}{2n}{∥Y-X\beta -K(\gamma ;Z)\alpha ∥}_{2}^{2}+\frac{1}{2}\lambda_{1}\alpha^{⊤}K\left(\gamma ;Z\right)\alpha +\lambda_{2}\rho \left(\gamma ;\delta \right). \end{array}$$

For fixed α, β and $\lambda_{1}$, minimizing the function in (A1) with respect to γ is equivalent to

(A2) $$\min_{\gamma }\left\{\frac{1}{2n}{∥\left(Y-X\beta -\frac{n}{2}\lambda_{1}\alpha \right)-K\left(\gamma ;Z\right)\alpha ∥}_{2}^{2}+\lambda_{2}\rho \left(\gamma ;\delta \right)\right\}.$$

### Appendix A.3. Proof of Theorem 1

With loss of the generality we use the penalty function for sparse group lasso but this proof can easily be modified for other penalty functions. Also, we fix $\lambda_{1}=\lambda_{2}=\delta =1$, and consider β∈R as well as set the design matrix X (or vector in this case) scaled to have norm 1. The case of $\beta \in R^{q}$ will follow along similar lines of arguments. Let $\gamma \in D_{3}$ with $D_{3}=\left\{\gamma :{∥\gamma ∥}_{1}\leq \frac{1}{2n}{∥Y∥}_{2}^{2}\right\}$. Define $f\left(\gamma \right)=∥K(\gamma ;Z)∥=\eta_{max}\left(K\left(\gamma ;Z\right)\right)\geq 0$, where $\eta_{max}\left(K\left(\gamma ;Z\right)\right)$ denotes the largest eigenvalue of K(γ;Z) with the operator norm (the norm of K(γ;Z)) defined in its usual way $∥K(\gamma ;Z)∥=sup\left\{{∥K(\gamma ;Z)x∥}_{2}^{2}:{∥x∥}_{2}^{2}=1\right\}$. Since $D_{3}$ is compact and K(γ;Z) is continuous with respect to γ it achieves its maximum over $D_{3}$. Thus, we define $\eta^{★}=sup_{\gamma \in D_{3}}f\left(\gamma \right)\geq 0$. Define $D_{2}=\left\{\beta :|\beta |\leq \left(1+\eta^{★}\right){∥Y∥}_{2}\right\}$, where the upper bound is denoted by $b^{★}=\left(1+\eta^{★}\right){∥Y∥}_{2}\geq 0.$ Moreover, define $D_{1}=\left\{\alpha :{∥\alpha ∥}_{2}\leq \sqrt{n}\left({∥Y∥}_{2}+b^{★}\right)\right\}.$

Since $D_{1},D_{2}$ and $D_{3}$ are compact there exists a $\left(\alpha^{★},\beta^{★},\gamma^{★}\right)$ such that $J_{2}\left(\alpha^{★},\beta^{★},\gamma^{★}\right)\leq J_{2}\left(\alpha ,\beta ,\gamma \right)$ for all $\left(\alpha ,\beta ,\gamma \right)\in D_{1}\times D_{2}\times D_{3}$. Note that $J_{2}\left(0,0,0\right)=\frac{1}{2n}{∥Y∥}_{2}^{2}$ and $\left(0,0,0\right)\in D_{1}\times D_{2}\times D_{3}$. We claim that $\left(\alpha^{★},\beta^{★},\gamma^{★}\right)$ is a global minimizer, which is proved below by contradiction.

Suppose that there exists $\left(\tilde{\alpha },\tilde{\beta },\tilde{\gamma }\right)\notin D_{1}\times D_{2}\times D_{3}$ where $J_{2}\left(\tilde{\alpha },\tilde{\beta },\tilde{\gamma }\right)<J_{2}\left(\alpha^{★},\beta^{★},\gamma^{★}\right)$. We must have that $\tilde{\gamma }\in D_{3}$; if not, we have $J_{2}\left(\tilde{\alpha },\tilde{\beta },\tilde{\gamma }\right)\geq {∥\tilde{\gamma }∥}_{1}\geq J_{2}\left(0,0,0\right)\geq J_{2}\left(\alpha^{★},\beta^{★},\gamma^{★}\right)$. Let $q_{1},\cdot \cdot \cdot ,q_{n}$ be the orthonormal vectors of $K(\tilde{\gamma };Z)$ with its associated eigenvalues $\eta_{1}\geq \cdots \geq \eta_{n}\geq 0$. We can write out $\tilde{\alpha },X,Y$ in terms of these basis functions where $\tilde{\alpha }=\sum_{i=1}^{n}<\tilde{\alpha },q_{i}>q_{i}$, $Y=\sum_{i=1}^{n}<Y,q_{i}>q_{i}$ and $X=\sum_{i=1}^{n}<X,q_{i}>q_{i}$. Let $C_{i}^{\tilde{\alpha }}=<\tilde{\alpha },q_{i}>$, $C_{i}^{Y}=<Y,q_{i}>$ and $C_{i}^{X}=<X,q_{i}>$. It follows that

$$J_{2}\left(\tilde{\alpha },\tilde{\beta },\tilde{\gamma }\right)\geq \frac{1}{2n}{∥\sum_{i=1}^{n}C_{i}^{Y}q_{i}-\sum_{i=1}^{n}C_{i}^{X}\tilde{\beta }q_{i}-\sum_{i=1}^{n}C_{i}^{\tilde{\alpha }}\eta_{i}q_{i}∥}_{2}^{2}+\frac{1}{2}\sum_{i=1}^{n}{\left(C_{i}^{\tilde{\alpha }}\right)}^{2}\eta_{i},$$

which is equal to $\frac{1}{2n}\sum_{i=1}^{n}{\left(C_{i}^{Y}-C_{i}^{X}\tilde{\beta }-C_{i}^{\tilde{\alpha }}\eta_{i}\right)}^{2}+\frac{1}{2}\sum_{i=1}^{n}{\left(C_{i}^{\tilde{\alpha }}\right)}^{2}\eta_{i}$. We can minimize the above objective function with respect to $C_{i}^{\tilde{\alpha }}$ and $\tilde{\beta }$. First, note that for any $\eta_{i}=0$ we can let $C_{i}^{\tilde{\alpha }}=0$ as it will not affect the expression above. It is sufficient to consider $\eta_{i}>0$. Taking the first derivative and setting it equal to zero, we obtain the score equations the minimizer must satisfy, for our minimum $\tilde{\beta }$ and $C_{i}^{\tilde{\alpha }}$

(A3) $$\beta =\sum_{i=1}^{n}C_{i}^{X}\left(C_{i}^{Y}-C_{i}^{\tilde{\alpha }}\eta_{i}\right)$$

(A4) $$C_{i}^{\tilde{\alpha }}=\frac{1}{n+\eta_{i}}\left(C_{i}^{Y}-C_{i}^{X}\tilde{\beta }\right).$$

In the above derivation we used the fact that $1={∥X∥}_{2}^{2}=\sum_{i=1}^{n}{\left(C_{i}^{X}\right)}^{2}$. Plugging (A4) into (A3), we obtain

(A5) $$\beta =\frac{\sum_{i=1}^{n}C_{i}^{X}C_{i}^{Y}\left(1-\frac{\eta_{i}}{n+\eta_{i}}\right)}{1-\sum_{i=1}^{n}{\left(C_{i}^{X}\right)}^{2}\frac{\eta_{i}}{n+\eta_{i}}}.$$

It follows that

$$\beta \leq \frac{\sum_{i=1}^{n}|C_{i}^{X}C_{i}^{Y}|}{1-\sum_{i=1}^{n}{\left(C_{i}^{X}\right)}^{2}\frac{\eta^{★}}{n+\eta^{★}}}\leq \frac{{∥X∥}_{2}{∥Y∥}_{2}}{{∥X∥}_{2}^{2}\left(1-\frac{\eta^{★}}{n+\eta^{★}}\right)}\leq \frac{{∥Y∥}_{2}}{\left(1-\frac{\eta^{★}}{1+\eta^{★}}\right)}=b^{★}.$$

Thus, the β that minimizes $J_{2}$ for a given $\gamma \in D_{3}$ is in $D_{2}$. Also, (A4) implies that $|C_{i}^{\tilde{\alpha }}|\leq \left({∥Y∥}_{2}+{∥X∥}_{2}{∥\beta ∥}_{2}\right)$; consequently, the optimal α for the given $\tilde{\gamma }\in D_{3}$ and $\beta \in D_{2}$ that minimizes $J_{2}$ satisfies ${∥\alpha ∥}_{2}\leq \sqrt{n}\left({∥Y∥}_{2}+b^{★}\right)$. As a result, $\alpha \in D_{2}$. This suggests that for any $\left(\tilde{\alpha },\tilde{\beta },\tilde{\gamma }\right)\notin D_{1}\times D_{2}\times D_{3}$ we can find an $\left(\alpha ,\beta ,\gamma \right)\in D_{1}\times D_{2}\times D_{3}$ such that $J_{2}\left(\tilde{\alpha },\tilde{\beta },\tilde{\gamma }\right)\geq J_{2}\left(\alpha ,\beta ,\gamma \right)$.

### Appendix A.4. Proof of Theorem 2

By Lemma 8.4 on page 129 in [32], Assumptions 1, 2, and 3 imply:

(A6) $$P\left(\underset{b\in B}{\sup}\frac{\frac{1}{\sqrt{n}}\left|\sum_{i=1}^{n}\epsilon_{i}b\left(z_{i}\right)\right|}{{∥b∥}_{P_{n}}^{1-\psi }}\geq T\right)\leq c\exp\left(-\frac{T^{2}}{c^{2}}\right),\;T\geq c$$

where the constant *c* is dependent on $C_{1},C_{2},C_{3},C_{4},$ and ψ. It follows that

(A7) $$\underset{b\in B}{\sup}\frac{\frac{1}{\sqrt{n}}\left|\sum_{i=1}^{n}\epsilon_{i}b\left(z_{i}\right)\right|}{{∥b∥}_{P_{n}}^{1-\psi }}=O_{p}\left(1\right).$$

Therefore, for any $h\in H_{K}$ and a scaling map function Γ∈A, we obtain

(A8) $$\frac{\sqrt{n}{\left(\epsilon ,h\circ \Gamma -h_{0}\circ \Gamma_{0}\right)}_{n}{\left({∥h∥}_{H_{K}}^{2}+{∥h_{0}∥}_{H_{K}}^{2}+{∥\Gamma ∥}_{SGL}^{2}+{∥\Gamma_{0}∥}_{SGL}^{2}\right)}^{-\psi }}{{∥h\circ \Gamma -h_{0}\circ \Gamma_{0}∥}_{P_{n}}^{1-\psi }}=O_{p}\left(1\right).$$

For our estimators, $\hat{h}$ and $\hat{\Gamma }$, it is easy to see that

(A9) $$\begin{array}{cc} {\left(\epsilon ,\hat{h}\circ \hat{\Gamma }-h_{0}\circ \Gamma_{0}\right)}_{n}= & \\ O_{p}\left(n^{-\frac{1}{2}}\right){∥\hat{h}\circ \hat{\Gamma }-h_{0}\circ \Gamma_{0}∥}_{n}^{1-\psi } & {\left({∥\hat{h}∥}_{H_{K}}^{2}+{∥h_{0}∥}_{H_{K}}^{2}+{∥\hat{\Gamma }∥}_{SGL}^{2}+{∥\Gamma_{0}∥}_{SGL}^{2}\right)}^{\psi }. \end{array}$$

From (A9), we obtain the following inequality:

(A10) $$\begin{array}{cc} \; & {∥\hat{h}\circ \hat{\Gamma }-h_{0}\circ \Gamma_{0}∥}_{n}^{2}+\lambda_{1}{∥\hat{h}∥}_{H_{K}}^{2}+\lambda_{2}{∥\hat{\Gamma }∥}_{SGL}^{2}\leq \\ \; & O_{p}\left(n^{-\frac{1}{2}}\right){∥\hat{h}\circ \hat{\Gamma }-h_{0}\circ \Gamma_{0}∥}_{n}^{1-\psi }{\left({∥\hat{h}∥}_{H_{K}}^{2}+{∥h_{0}∥}_{H_{K}}^{2}+{∥\hat{\Gamma }∥}_{SGL}^{2}+{∥\Gamma_{0}∥}_{SGL}^{2}\right)}^{\psi }\\ \; & +\lambda_{1}{∥h_{0}∥}_{H_{K}}^{2}+\lambda_{2}{∥\Gamma_{0}∥}_{SGL}^{2}. \end{array}$$

We require $\lambda_{1}=O_{p}\left(1\right)\lambda_{2}$, namely $\lambda_{2}$ and $\lambda_{1}$ go to zero at the same rate. We will show at the end of the proof what happens if they are not of the same order. Therefore, without loss of generality, we set $\lambda_{1}=\lambda_{2}$, denoted by λ. In what follows, we divide (A10) into two cases.

Case 1: Suppose that

$$\begin{array}{c} O_{p}\left(n^{-\frac{1}{2}}\right){∥\hat{h}\circ \hat{\Gamma }-h_{0}\circ \Gamma_{0}∥}_{n}^{1-\psi }{\left({∥\hat{h}∥}_{H_{K}}^{2}+{∥h_{0}∥}_{H_{K}}^{2}+{∥\hat{\Gamma }∥}_{SGL}^{2}+{∥\Gamma_{0}∥}_{SGL}^{2}\right)}^{\psi }\\ \geq \lambda \left({∥h_{0}∥}_{H_{K}}^{2}+{∥\Gamma_{0}∥}_{SGL}^{2}\right). \end{array}$$

In this case, we have

(A11) $$\begin{array}{cc} \; & {∥\hat{h}\circ \hat{\Gamma }-h_{0}\circ \Gamma_{0}∥}_{n}^{2}+\lambda \left({∥\hat{h}∥}_{H_{K}}^{2}+{∥\hat{\Gamma }∥}_{SGL}^{2}\right)\leq \\ \; & O_{p}\left(n^{-\frac{1}{2}}\right){∥\hat{h}\circ \hat{\Gamma }-h_{0}\circ \Gamma_{0}∥}_{n}^{1-\psi }{\left({∥\hat{h}∥}_{H_{K}}^{2}+{∥h_{0}∥}_{H_{K}}^{2}+{∥\hat{\Gamma }∥}_{SGL}^{2}+{∥\Gamma_{0}∥}_{SGL}^{2}\right)}^{\psi }. \end{array}$$

Above (A11) is further discussed separately in two sub-cases.

Case 1a: If ${∥h_{0}∥}_{H_{K}}^{2}+{∥\Gamma_{0}∥}_{SGL}^{2}\leq {∥\hat{h}∥}_{H_{K}}^{2}+{∥\hat{\Gamma }∥}_{SGL}^{2}$, then we have

(A12) $$\begin{array}{c} {∥\hat{h}\circ \hat{\Gamma }-h_{0}\circ \Gamma_{0}∥}_{n}^{2}+\lambda \left({∥\hat{h}∥}_{H_{K}}^{2}+{∥\hat{\Gamma }∥}_{SGL}^{2}\right)\leq \\ O_{p}\left(n^{-\frac{1}{2}}\right){∥\hat{h}\circ \hat{\Gamma }-h_{0}\circ \Gamma_{0}∥}_{n}^{1-\psi }{\left({∥\hat{h}∥}_{H_{K}}^{2}+{∥\hat{\Gamma }∥}_{SGL}^{2}\right)}^{\psi }. \end{array}$$

Therefore,

(A13) $$\begin{array}{c} {\left({∥\hat{h}∥}_{H_{K}}^{2}+{∥\hat{\Gamma }∥}_{SGL}^{2}\right)}^{\psi }\leq O_{p}\left(n^{-\frac{\psi }{2(1-\psi )}}\right){∥\hat{h}\circ \hat{\Gamma }-h_{0}\circ \Gamma_{0}∥}_{n}^{\psi }\lambda^{-\frac{\psi }{1-\psi }}. \end{array}$$

It follows that

(A14) $$\begin{array}{c} {∥\hat{h}\circ \hat{\Gamma }-h_{0}\circ \Gamma_{0}∥}_{n}=O_{p}\left(n^{-\frac{1}{2(1-\psi )}}\right)O_{p}\left(\lambda^{-\frac{\psi }{1-\psi }}\right),\\ {∥\hat{h}∥}_{H_{K}}^{2}+{∥\hat{\Gamma }∥}_{SGL}^{2}=O_{p}\left(n^{-\frac{1}{1-\psi }}\right)O_{p}\left(\lambda^{-\frac{1+\psi }{1-\psi }}\right). \end{array}$$

Case 1b: If ${∥h_{0}∥}_{H_{K}}^{2}+{∥\Gamma_{0}∥}_{SGL}^{2}\geq {∥\hat{h}∥}_{H_{K}}^{2}+{∥\hat{\Gamma }∥}_{SGL}^{2}$, then:

$${∥\hat{h}∥}_{H_{K}}^{2}+{∥\hat{\Gamma }∥}_{SGL}^{2}=O_{p}\left({∥h_{0}∥}_{H_{K}}^{2}+{∥\Gamma_{0}∥}_{SGL}^{2}\right)O_{p}\left(1\right).$$

Therefore,

$${∥\hat{h}\circ \hat{\Gamma }-h_{0}\circ \Gamma_{0}∥}_{n}=O_{p}\left(n^{-\frac{1}{2(1+\psi )}}\right){\left({∥h_{0}∥}_{H_{K}}^{2}+{∥\Gamma ∥}_{SGL}^{2}]\right)}^{\frac{\psi }{1+\psi }}.$$

Consequently, we obtain

(A15) $$\begin{array}{c} {∥\hat{h}\circ \hat{\Gamma }-h_{0}\circ \Gamma_{0}∥}_{n}=O_{p}\left(n^{-\frac{1}{2(1-\psi )}}\right)O_{p}\left(\lambda^{-\frac{\psi }{1-\psi }}\right),\\ {∥\hat{h}∥}_{H_{K}}^{2}+{∥\hat{\Gamma }∥}_{SGL}^{2}=O_{p}\left(n^{-\frac{1}{1-\psi }}\right)O_{p}\left(\lambda^{-\frac{1+\psi }{1-\psi }}\right). \end{array}$$

Both terms in (A15) are the same rates as those in (A14).

Case 2: Suppose that

$$\begin{array}{c} O_{p}\left(n^{-\frac{1}{2}}\right){∥\hat{h}\circ \hat{\Gamma }-h_{0}\circ \Gamma_{0}∥}_{n}^{1-\psi }{\left({∥\hat{h}∥}_{H_{K}}^{2}+{∥h_{0}∥}_{H_{K}}^{2}+{∥\hat{\Gamma }∥}_{SGL}^{2}+{∥\Gamma_{0}∥}_{SGL}^{2}\right)}^{\psi }\\ \leq \lambda ({∥h_{0}∥}_{H_{K}}^{2}+{∥\Gamma_{0}∥}_{SGL}^{2}). \end{array}$$

Then, we have

$${∥\hat{h}\circ \hat{\Gamma }-h_{0}\circ \Gamma_{0}∥}_{n}^{2}+\lambda \left({∥\hat{h}∥}_{H_{K}}^{2}+{∥\hat{\Gamma }∥}_{SGL}^{2}\right)\leq 2\lambda \left({∥h_{0}∥}_{H_{K}}^{2}+{∥\Gamma_{0}∥}_{SGL}^{2}\right).$$

This implies that

(A16) $$\begin{array}{c} {∥\hat{h}\circ \hat{\Gamma }-h_{0}\circ \Gamma_{0}∥}_{n}=O_{p}\left(\lambda^{\frac{1}{2}}\right){\left({∥h_{0}∥}_{H_{K}}^{2}+{∥\Gamma_{0}∥}_{SGL}^{2}\right)}^{\frac{1}{2}},\\ {∥\hat{h}∥}_{H_{K}}^{2}+{∥\hat{\Gamma }∥}_{SGL}^{2}=O_{p}\left(1\right)\left({∥h_{0}∥}_{H_{K}}^{2}+{∥\Gamma_{0}∥}_{SGL}^{2}\right). \end{array}$$

In order to make (A14) and (A16) have the same rates we first equate the two term $O_{p}\left(\lambda^{\frac{1}{2}}\right){\left({∥h∥}_{H_{K}}^{2}+{∥\Gamma ∥}_{SGL}^{2}\right)}^{\frac{1}{2}}$ and $O_{p}\left(n^{-\frac{1}{2(1-\psi )}}\right)O_{p}\left(\lambda^{-\frac{\psi }{1-\psi }}\right)$, and then solve for a common λ. The solution is given as follows:

$$\lambda^{-1}=n^{\frac{1}{1+\psi }}{\left({∥h∥}_{H_{K}}^{2}+{∥\Gamma ∥}_{SGL}^{2}\right)}^{\frac{1-\psi }{1+\psi }}.$$

Under this λ value we obtain that (A14)–(A16) as of the form:

(A17) $$\begin{array}{c} {∥\hat{h}\circ \hat{\Gamma }-h_{0}\circ \Gamma_{0}∥}_{n}=O_{p}\left(n^{-\frac{1}{2(1+\psi )}}\right){\left({∥h_{0}∥}_{H_{K}}^{2}+{∥\Gamma_{0}∥}_{SGL}^{2}\right)}^{\frac{\psi }{1+\psi }}, \end{array}$$

(A18) $$\begin{array}{c} {∥\hat{h}∥}_{H_{K}}^{2}+{∥\hat{\Gamma }∥}_{SGL}^{2}=O_{p}\left(1\right)\left({∥h_{0}∥}_{H_{K}}^{2}+{∥\Gamma_{0}∥}_{SGL}^{2}\right). \end{array}$$

This completes the proof of Theorem 2.

Now we discuss the situation where the tuning parameters $\lambda_{1}$ and $\lambda_{2}$ are not of the same order. As seen blow, the selection consistency may not be guaranteed. Take Case 2 as an example. Suppose that

$$\begin{array}{c} O_{p}\left(n^{-\frac{1}{2}}\right){∥\hat{h}\circ \hat{\Gamma }-h_{0}\circ \Gamma_{0}∥}_{n}^{1-\psi }{\left({∥\hat{h}∥}_{H_{K}}^{2}+{∥h_{0}∥}_{H_{K}}^{2}+{∥\hat{\Gamma }∥}_{SGL}^{2}+{∥\Gamma_{0}∥}_{SGL}^{2}\right)}^{\psi }\\ \leq \lambda_{1}{∥h_{0}∥}_{H_{K}}^{2}+\lambda_{2}{∥\Gamma_{0}∥}_{SGL}^{2}. \end{array}$$

Let us consider two cases.

Case 2a: If $\lambda_{1}{∥h_{0}∥}_{H_{K}}^{2}\leq \lambda_{2}{∥\Gamma_{0}∥}_{SGL}^{2}$, following the same arguments above, we have

(A19) $$\begin{array}{c} {∥\hat{h}\circ \hat{\Gamma }-h_{0}\circ \Gamma_{0}∥}_{n}=O_{p}\left(\lambda_{2}^{\frac{1}{2}}\right){∥\Gamma_{0}∥}_{SGL}),\\ {∥\hat{h}∥}_{H_{K}}^{2}=O_{p}\left(\frac{\lambda_{2}}{\lambda_{1}}\right){∥\Gamma_{0}∥}_{SGL}^{2},\\ {∥\hat{\Gamma }∥}_{SGL}^{2}=O_{p}\left(1\right){∥\Gamma_{0}∥}_{SGL}^{2}. \end{array}$$

Case 2b: If $\lambda_{1}{∥h_{0}∥}_{H_{K}}^{2}\geq \lambda_{2}{∥\Gamma_{0}∥}_{SGL}^{2}$, then following the same logic as before:

(A20) $$\begin{array}{c} {∥\hat{h}\circ \hat{\Gamma }-h_{0}\circ \Gamma_{0}∥}_{n}=O_{p}\left(\lambda_{1}^{\frac{1}{2}}\right){∥h_{0}∥}_{H_{K}}),\\ {∥\hat{\Gamma }∥}_{SGL}^{2}=O_{p}\left(\frac{\lambda_{1}}{\lambda_{2}}\right){∥h_{0}∥}_{H_{K}}^{2},\\ {∥\hat{h}∥}_{H_{K}}^{2}=O_{p}\left(1\right){∥h_{0}∥}_{H_{K}}^{2}. \end{array}$$

Both terms involve $O_{p}\left(\frac{\lambda_{1}}{\lambda_{2}}\right)$ and $O_{p}\left(\frac{\lambda_{2}}{\lambda_{1}}\right)$, indicating that these two tuning parameters $\lambda_{1}$ and $\lambda_{2}$ should go to zero at the same rates. Moreover, we can think of our estimator $\hat{h}\circ \hat{\Gamma }$ as one operational object. See Appendix B for more details on this, which can further explain the need of one rate for the two penalties.

### Appendix A.5. Proof of Corollary 1

For convenience, we present the following lemma proved by [32] (on page 20).

**Lemma** **A1.**
*(Geer’s Lemma) A d dimensional ball of radius R, $B_{d}\left(R\right)$, in $R^{d}$ with Euclidean metric can be covered by ${\left(\frac{4R+\delta }{\delta }\right)}^{d}$ balls of radius δ.*

We have shown in the proof of Theorem 1 that the optimal γ vector is restricted to be within a ball of a radius that depends on the norm of Y. For the sake of simplicity let us confine our γ to be within a norm ball of radius 1, $\gamma \in G=\{\gamma :{∥\gamma ∥}_{2}^{2}\leq 1\}$. We then confine our set which we called A to be restricted to those γ, that is A={Γ:Γ(z)=γ∘z,γ∈G}. Since our $\gamma \in R^{s}$, we can use above Lemma A1 and cover our set A with $N_{1}={\left(\frac{4+\delta }{\delta }\right)}^{s}$ number of functions in the following sense. The ball of radius 1 in $R^{s}$ can be covered (using the Euclidean metric) by $\left\{\gamma_{1},\cdots \gamma_{N_{1}}\right\}$. Since there is a one to one relationship between the functions Γ and γ, take the set $\left\{\Gamma_{1},\ldots ,\Gamma_{N_{1}}\right\}$ and define the metric between some $\Gamma_{j}$ and $\Gamma_{k}$ in the set A as $d\left(\Gamma_{j},\Gamma_{k}\right)={∥\gamma_{j}-\gamma_{k}∥}_{2}$. Then, the set of functions $\left\{\Gamma_{1},\ldots ,\Gamma_{N_{1}}\right\}$ is a δ-covering for A under this metric with entropy *s* $log(\frac{4+\delta }{\delta })$. For each $\Gamma_{j}$ we have an induced RKHS, $H_{K\circ \Gamma_{j}}=\left\{h\circ \Gamma_{j}:h\in H_{K}\right\}$ with entropy no larger than that of $H_{K}$, which according to the assumption, has entropy $\leq A\delta^{-2\psi }$ for some ψ∈(0,1) and A∈R. Therefore, the covering number $N_{2}=N\left(\delta ,H_{K\circ \Gamma_{j}},P_{n}\right)\leq \exp\left\{A\delta^{-2\psi }\right\}$. This implies that for every $\Gamma_{j}$ there exists a set $\left\{h_{j_{1}}\circ \Gamma_{j},\cdots ,h_{j_{N_{2}}}\circ \Gamma_{j}\right\}$ such that for every $h\circ \Gamma_{j}\in H_{K\circ \Gamma_{j}}$ there exists an integer $i\in \{1,\ldots ,N_{2}\}$ we have ${∥h\circ \Gamma_{j}-h_{j_{i}}\circ \Gamma_{j}∥}_{P_{n}}\leq \delta$. Set B is essentially the union of the different Hilbert spaces of the form $H_{K\circ \Gamma }$. Under the setup, a natural estimate of the delta-covering number of this set would be approximately of size $N_{1}\times N_{2}$ where functions take the form of $\left\{h_{1_{1}}\circ \Gamma_{1},\cdots ,h_{1_{N_{2}}}\circ \Gamma_{1},\cdots ,h_{{N_{1}}_{1}}\circ \Gamma_{N_{1}},\cdots ,h_{{N_{1}}_{N_{2}}}\circ \Gamma_{N_{1}}\right\}$. In addition, we add $N_{2}$ functions from the set $\left\{h_{1}\circ \Gamma_{0},\cdots ,h_{N_{2}}\circ \Gamma_{0}\right\}$ where $\Gamma_{0}$ is the true $\Gamma_{0}$ (or one of the true $\Gamma_{0}$). Since $H_{K\circ \Gamma_{j}}$ is a Hilbert space for every *j*, if $h\circ \Gamma_{j}\in H_{K\circ \Gamma_{j}}$ so is $\frac{h\circ \Gamma_{j}}{{∥h∥}_{H_{K}}^{2}+{∥h_{0}∥}_{H_{K}}^{2}+{∥\Gamma_{j}∥}_{SGL}^{2}+{∥\Gamma_{0}∥}_{SGL}^{2}}$. We can simply ignore the denominator and substitute $\frac{h\circ \Gamma_{j}}{{∥h∥}_{H_{K}}^{2}+{∥h_{0}∥}_{H_{K}}^{2}+{∥\Gamma_{j}∥}_{SGL}^{2}+{∥\Gamma_{0}∥}_{SGL}^{2}}$ with $\tilde{h}\circ \Gamma_{j}\in H_{K\circ \Gamma_{j}}$ where $\tilde{h}=\frac{h}{{∥h∥}_{H_{K}}^{2}+{∥h_{0}∥}_{H_{K}}^{2}+{∥\Gamma_{j}∥}_{SGL}^{2}+{∥\Gamma_{0}∥}_{SGL}^{2}}.$

We now prove Corollary 1.

**Proof.** Set $M=\sup_{h}<\nabla h\left(z\right),\nabla h\left(z\right)>$ where the inner product is the standard Euclidean inner product. This is for a fixed z, or under the assumption that the gradient is uniformly bounded, we can take the $\sup_{h\in H_{K},z\in R^{s}}<\nabla h\left(z\right),\nabla h\left(z\right)>$. Let $N_{1}={\frac{4+\left(\frac{\delta }{3M^{\frac{1}{2}}}\right)}{\left(\frac{\delta }{3M^{\frac{1}{2}}}\right)}}^{s}$ which is the number of balls needed to provide a $\left(\frac{\delta }{3M^{\frac{1}{2}}}\right)$ covering for a norm 1 ball in $R^{s}$. Let $N_{2}=\exp\left\{\left(A{\left(\frac{\delta }{3}\right)}^{-2\psi }\right)\right\}$ which is the covering number needed to provide a $\frac{\delta }{3}$ cover of our space $H_{K}$. Let:

$$\begin{array}{cc} \; & \tilde{\hat{h}}\circ \hat{\Gamma }-{\tilde{h}}_{0}\circ \Gamma_{0}=\\ \; & \frac{\hat{h}\circ \hat{\Gamma }}{{∥\hat{h}∥}_{H_{K}}^{2}+{∥h_{0}∥}_{H_{K}}^{2}+{∥\hat{\Gamma }∥}_{SGL}^{2}+{∥\Gamma_{0}∥}_{SGL}^{2}}-\frac{h_{0}\circ \Gamma_{0}}{{∥\hat{h}∥}_{H_{K}}^{2}+{∥h_{0}∥}_{H_{K}}^{2}+{∥\hat{\Gamma }∥}_{SGL}^{2}+{∥\Gamma_{0}∥}_{SGL}^{2}} \end{array}$$

be an arbitrary function in the set B. There exists a $\Gamma_{j}$ where $j\in \{1,\ldots ,N_{1}\}$ such that $d\left(\Gamma_{j},\hat{\Gamma }\right)\leq \frac{\delta }{3\max_{i=1,\cdots ,n}{∥z_{i}∥}_{2}\sqrt{M}}$, and there exists an *i* where $i\in \{1,\ldots ,N_{2}\}$ such that ${∥\tilde{\hat{h}}\circ \Gamma_{j}-h_{j_{i}}\circ \Gamma_{j}∥}_{P_{n}}\leq \frac{\delta }{3}$. Similarly, there exists a $t\in \{1,\ldots ,N_{2}\}$ such that ${∥{\tilde{h}}_{0}\circ \Gamma_{0}-h_{t}\circ \Gamma_{0}∥}_{P_{n}}\leq \frac{\delta }{3}$. We construct our approximating function of $\tilde{\hat{h}}\circ \hat{\Gamma }-{\tilde{h}}_{0}\circ \Gamma_{0}$ as $h_{j_{i}}\circ \Gamma_{j}-h_{t}\circ \Gamma_{0}$. We now show that this function is within δ of our arbitrary function $\tilde{\hat{h}}\circ \hat{\Gamma }-{\tilde{h}}_{0}\circ \Gamma_{0}$. Applying the mean value theorem for multivariate functions, $\tilde{\hat{h}}\circ \hat{\Gamma }\left(z\right)=\tilde{\hat{h}}\circ \Gamma_{j}\left(z\right)+\nabla \tilde{\hat{h}}\left(C\left(z\right)\right)\left(\hat{\Gamma (z)}-\Gamma_{j}\left(z\right)\right)$, we have:

$$\begin{array}{cc} \|(\tilde{\hat{h}}\circ & \hat{\Gamma }-{\tilde{h}}_{0}\circ \Gamma_{0})-\left(h_{j_{i}}\circ \Gamma_{j}-h_{t}\circ \Gamma_{0}\right){\|}_{P_{n}}\\ & \leq {∥\tilde{\hat{h}}\circ \hat{\Gamma }-h_{j_{i}}\circ \Gamma_{j}∥}_{P_{n}}+{∥{\tilde{h}}_{0}\circ \Gamma_{0}-h_{t}\circ \Gamma_{0}∥}_{P_{n}}\\ & \leq {∥\tilde{\hat{h}}\circ \hat{\Gamma }-h_{j_{i}}\circ \Gamma_{j}∥}_{P_{n}}+\frac{\delta }{3}\\ & ={∥\tilde{\hat{h}}\circ \Gamma_{j}-h_{j_{i}}\circ \Gamma_{j}+\nabla \tilde{\hat{h}}\left(C\left(\cdot \right)\right)\left(\hat{\Gamma }-\Gamma_{j}\right)∥}_{P_{n}}+\frac{\delta }{3} \end{array}$$

where vector $z\in R^{s}$ lies in the segment from $\gamma_{j}\circ z$ and $\hat{\gamma }\circ z$, and C(·) is an unknown function that maps from $R^{s}$ into $R^{s}$ that allows for the formula to hold. Continuing our chain of inequalities, we obtain:

$$\begin{array}{c} {∥\tilde{\hat{h}}\circ \Gamma_{j}-h_{j_{i}}\circ \Gamma_{j}+\nabla \tilde{\hat{h}}\left(C\left(\cdot \right)\right)\left(\hat{\Gamma }-\Gamma_{j}\right)∥}_{P_{n}}+\frac{\delta }{3}\leq \\ {∥\nabla \tilde{\hat{h}}\left(C\left(\cdot \right)\right)\left(\hat{\Gamma }-\Gamma_{j}\right)∥}_{P_{n}}+\frac{\delta }{3}+\frac{\delta }{3}=\\ \sqrt{\frac{1}{n}\sum_{i=1}^{n}{\left(\nabla \tilde{\hat{h}}\left(C\left(z_{i}\right)\right)\left(\hat{\Gamma }\left(z_{i}\right)-\Gamma_{j}\left(z_{i}\right)\right)\right)}^{2}}+\frac{\delta }{3}+\frac{\delta }{3}\leq \\ \sqrt{\frac{1}{n}\sum_{i=1}^{n}M{∥\hat{\gamma }\circ z_{i}-\gamma_{j}\circ z_{i}∥}_{2}^{2}}+\frac{\delta }{3}+\frac{\delta }{3}\leq \\ \sqrt{M{\left(\frac{\delta }{3\max_{i=1,\cdots ,n}{∥z_{i}∥}_{2}\sqrt{M}}\right)}^{2}\max_{i=1,\cdots ,n}{∥z_{i}∥}_{2}^{2}}+\frac{\delta }{3}+\frac{\delta }{3}=\\ \frac{\delta }{3}+\frac{\delta }{3}+\frac{\delta }{3}=\delta . \end{array}$$

Therefore, to provide a δ cover we need $N_{1}\times N_{2}+N_{2}$ number of functions or:

$$\begin{array}{c} \exp\left\{\left(A{\left(\frac{\delta }{3}\right)}^{-2\psi }\right)\right\}{\left(\frac{4+\left(\frac{\delta }{3M^{\frac{1}{2}}}\right)}{\left(\frac{\delta }{3M^{\frac{1}{2}}}\right)}\right)}^{s}+\exp\left\{\left(A{\left(\frac{\delta }{3}\right)}^{-2\psi }\right)\right\}=\\ \exp\left\{\tilde{A}\delta^{-2\psi }\right\}{\left(\frac{C+\delta }{\delta }\right)}^{s}+\exp\left\{\tilde{A}\delta^{-2\psi }\right\}, \end{array}$$

where $\tilde{A}=\frac{A}{3^{-2\psi }}$ and $C=12M^{\frac{1}{2}}$. Taking the log we see the entropy is $\leq \tilde{A}\delta^{-2\psi }+\log\left({\left(\frac{C+\delta }{\delta }\right)}^{s}+1\right)$ which is of the same order as $\leq \tilde{A}\delta^{-2\psi }$ (the log term is dominated by the first term). Therefore a sufficient (but not necessary) condition for our set B to have the same entropy as that of the original RKHS $H_{K}$ is for the $\sup_{h}<\nabla h\left(z\right),\nabla h\left(z\right)>$ to be bounded. Having bounded derivatives is reasonable for any RKHS since every RKHS satisfies the Lipschitz condition of the form:

$$\begin{array}{c} |h\left(X\right)-h\left(Y\right)|=|<h,K_{X}>-<h,K_{Y}>|\leq {∥h∥}_{H_{K}}<K_{X},K_{Y}{>}^{\frac{1}{2}}={∥h∥}_{H_{K}}d\left(X,Y\right), \end{array}$$

where the distance metric in $R^{s}$ is defined as $d{\left(X,Y\right)}^{2}=K\left(X,X\right)-2K\left(X,Y\right)+K\left(Y,Y\right)$. If we restrict our functions in the RKHS of norm ≤C for some constant *C* then we have a universal Lipschitz constant *C* to ensure bounded derivatives. □

## Appendix B. Discussion about the FKMR Estimator

We introduce γ as a way of performing variable selection on our vector of FPC features. We want to illustrate this technical trick with some concrete examples and discuss identifiability issues with the resulting estimator. There are two ways of looking at the estimation of the unknown functions $h_{0}$ and $\Gamma_{0}$. The first way is to view our feature vector, z, as being related to the dependent variable *y* through the composite function h∘Γ, as explained in Section 4. The second and equivalent way is to view our features as unknown. The true features take the form of γ∘z, where in this case the ∘ denotes the Hadamard product. We are given z and need to estimate the “true" features γ∘z. In addition, we need to estimate the relationship between γ∘z and *y*, which is done through the function $h\in H_{K}$.

The first way is to estimate the function $h_{0}\circ \Gamma_{0}$. The function belongs to the RKHS $H_{K\circ \Gamma }$. We essentially consider many different function spaces to construct our estimator. The intersection between the function spaces is not necessarily empty, implying that our estimator may not be unique. We proceed this discussion more formally. Let $K:R^{s}\times R^{s}\mapsto R$ be a positive definite function. Let $\Gamma :R^{s}\mapsto R^{s}$. We define $K\circ \Gamma :R^{s}\times R^{s}\mapsto R$ as the function given by K∘Γ(s,t)=K(Γ(s),Γ(t)). This new function, K∘Γ is positive definite. There is a relationship between the original RKHS, $H_{K}$ and the new RKHS, $H_{K\circ \Gamma }$. This results in $H_{K\circ \Gamma }=\left\{h\circ \Gamma :h\in H_{K}\right\}$. For any vector $u\in H_{K\circ \Gamma }$, we have that ${∥u∥}_{H_{K\circ \Gamma }}=inf\left\{{∥h∥}_{H_{K}}:u=h\circ \Gamma \right\}$. In general, $H_{K\circ \Gamma }\neg \subset H_{K}$. In (5), we take the norm with respect to the original space $H_{K}$. Our iterative procedure essentially presents the second way in which the true features are unknown, whereas our theoretical arguments are justified through the first way. Given the knowledge of the features (which translates to fixing a γ), we are confined to just one RKHS, $H_{K}$. Take the linear kernel, $K\left(x_{1},x_{2}\right)=x_{1}^{⊤}x_{2}$ as an example. Suppose the truth is that *y* is related to a one-dimensional feature $z_{0}$ through the following formulation: $y=h_{0}\left(z_{0}\right)+\varepsilon$ where $h_{0}\in H_{K_{1}}$, where $K_{1}$ is the kernel that maps from R×R↦R. Therefore, if we knew the feature $z_{1}$, we would proceed to optimize (6) using the standard LSKM. However, when each *y* is associated with a two-dimensional vector $z=(z_{1},z_{2})$, where $z_{2}$ is a “noisy” feature and unrelated to *y*. Suppose that *a priori* we do not know this information. Typically we use a model $y=h(z_{1},z_{2})+\varepsilon$ where $h\in H_{K}$, where K is the kernel that maps from $R^{2}\times R^{2}\mapsto R$. In this case, we introduce our γ vector $\left(\gamma_{1},\gamma_{2}\right)$ and formulate $y=h(\gamma_{1}z_{1},\gamma_{2}z_{2})+\epsilon$. All functions, *h* in the space $H_{K}$, are of the form $h\left(z\right)=x^{⊤}z$ for some two-dimensional vector $x=(x_{1},x_{2})$. There is a one-to-one relationship between *h* and x. The true function, $h_{0}$, has an associated real number *c* where $h_{1}\left(z_{1}\right)=cz_{1}$. We can recover $h_{1}\in H_{K_{1}}$ from our estimation of *h* and γ if we set γ=(1,0) and x=(c,★), where "★" is any real number. Equivalently, we can recover $h_{1}$ under γ=(1,1) where x=(c,0). There are many functions that may recover the original function in the RKHS corresponding to the linear space kernel. Formulating our problem in the first way, through function composition, we can estimate $\Gamma_{0}$ with the γ being (1,0) or (1,1).

We can now see that in the intersection between $H_{K\circ \Gamma_{1}}$ and $H_{K\circ \Gamma_{2}}$, where $\Gamma_{1}$ has associated $\gamma_{1}=\left(1,0\right)$ and $\Gamma_{2}$ has associated $\gamma_{2}=\left(1,1\right)$, lies our estimate of $h_{1}$. In truth, for the linear space RKHS, there is no need to apply our method since $h_{0}\in H_{K_{1}}$ can be estimated directly from the larger space $H_{K}$ where we set $h\left(z\right)=x^{⊤}z$ where x=(c,0). We can never hope to have variable selection consistency nor can we hope to have identifiability of our estimator for these types of spaces. However, from a goodness-of-fit standpoint, we are able to do just as good a job with many types of function compositions. Our hope is that we can glean some variable selection by penalizing the γ vector with the ρ(γ;δ) term which, going back to the above scenario, should give preference to γ=(1,0) over γ=(1,1). For the RKHS associated with the Gaussian Kernel, the “larger dimensional space”, a Gaussian Kernel mapping from higher dimensions, does not necessarily contain the functions from a “lower dimensional space”, a Gaussian Kernel mapping from lower dimensions. However through the introduction of the γ transformation of the features, we can recover the equivalent functions of the "lower dimensional space”.
